# Immunological Changes in Pregnancy and Prospects of Therapeutic Pla-Xosomes in Adverse Pregnancy Outcomes

**DOI:** 10.3389/fphar.2022.895254

**Published:** 2022-04-20

**Authors:** Himadri Devvanshi, Rohit Kachhwaha, Anima Manhswita, Shinjini Bhatnagar, Pallavi Kshetrapal

**Affiliations:** ^1^ Maternal and Child Health, Translational Health Science and Technology Institute, Faridabad, India; ^2^ School of Agriculture and Food Science, The University of Queensland, Brisbane, QLD, Australia

**Keywords:** pregnancy, pla-xosomes, cancer, exosomes, adverse pregnancy outcome, immune exhaustion, immune-therapy

## Abstract

Stringent balance of the immune system is a key regulatory factor in defining successful implantation, fetal development, and timely parturition. Interference in these primary regulatory mechanisms, either at adolescence or prenatal state led to adverse pregnancy outcomes. Fertility restoration with the help of injectable gonadotrophins/progesterone, ovulation-inducing drugs, immunomodulatory drugs (corticosteroids), and reproductive surgeries provides inadequate responses, which manifest its own side effects. The development of a potential diagnostic biomarker and an effectual treatment for adverse pregnancy outcomes is a prerequisite to maternal and child health. Parent cell originated bi-layered-intraluminal nano-vesicles (30–150 nm) also known as exosomes are detected in all types of bodily fluids like blood, saliva, breast milk, urine, etc. Exosomes being the most biological residual structures with the least cytotoxicity are loaded with cargo in the form of RNAs (miRNAs), proteins (cytokines), hormones (estrogen, progesterone, etc.), cDNAs, and metabolites making them chief molecules of cell-cell communication. Their keen involvement in the regulation of biological processes has portrayed them as the power shots of cues to understand the disease’s pathophysiology and progression. Recent studies have demonstrated the role of immunexosomes (immunomodulating exosomes) in maintaining unwavering immune homeostasis between the mother and developing fetus for a healthy pregnancy. Moreover, the concentration and size of the exosomes are extensively studied in adverse pregnancies like preeclampsia, gestational diabetes mellitus (GDM), and preterm premature rupture of membrane (pPROMs) as an early diagnostic marker, thus giving in-depth information about their pathophysiology. Exosomes have also been engineered physically as well as genetically to enhance their encapsulation efficiency and specificity in therapy for cancer and adverse pregnancies. Successful bench to bedside discoveries and interventions in cancer has motivated developmental biologists to investigate the role of immunexosomes and their active components. Our review summarizes the pre-clinical studies for the use of these power-shots as therapeutic agents. We envisage that these studies will pave the path for the use of immunexosomes in clinical settings for reproductive problems that arise due to immune perturbance in homeostasis either at adolescence or prenatal state.

## Introduction

The semi-allogenic fetus develops and resides within the mother’s womb, causing a series of physiological, structural, organismal changes in her body. These profound changes take place proximally in the endometrium and the uterine cavity to protect the fetus from rejection via modulation of the maternal immune system and structural remodeling to provide better nutrition for the growing fetus. Distally the informed changes in maternal physiology are an adaptation process in order to prepare the mother for the rest of the gestational journey. The endocrine signals (progesterone, estrogen, human chorionic gonadotrophin (hCG), genomic (miRNAs), and metabolomic entities (lipids, amino acids, etc.) work in conjunction to progenerate the maternal immune system towards accepting the fetal antigens, which is a kind of stress test for the mother (Bukovsky et al., 2003; Li et al., 2004; Mulac-Jericevic and Conneely, 2004; Rolle et al., 2013; Jabrane-Ferrat, 2019). The fetoplacental communication resembles a webbed structure with every node impersonating an immune cell, required to maintain equilibrium among all cells in the unit. The maternal immune system is renovated, providing a suppressive immune niche for fetal survival, thus establishing a crucial feto-maternal immune crosstalk. Interestingly in cancer, a similar mechanism of reconditioning the immune system for favorable changes is very well studied (Costanzo et al., 2018). Cancer progression is thus a phenomenon of forced changes and has similarities with regulated fetal growth during pregnancy. The host and maternal immune system engage in a contest of strength towards producing a response against developing cancer and the fetus. Ultimately this response modulates the host and maternal immune system resulting in the establishment of cancer and sustenance of the fetus, respectively. This immunomodulation is effectively aided by the signals emanating within the bilayered-intraluminal nanovesicles, which work distally in maintaining the immune crosstalk for their stabilization (Salomon et al., 2014a). Discovered almost 40 years ago in 1989 (Trams et al., 1981; Pan and Johnstone, 1983), the extracellular vesicles named exosomes were characterized later as lipid-bilayered-intraluminal microvesicles (ILVs) (30–150 nm), yielded by invagination of multivesicular bodies (MVBs) derived from endosomes during stress response or for cell-cell communication (Harding et al., 1984). Exosomes are decisive in an aspect because they encapsulate regulatory signals of cellular behavior. Demonstrated in the database, over 9,690 kinds of proteins, more than 3,300 varieties of mRNAs, and 1,010 different types of lipids can exist in an exosome depending upon its origin (Keerthikumar et al., 2016; Kurian and Modi, 2019). Studies have represented that the exosomes are extensively involved in feto-maternal communication facilitating embryo implantation, trophoblast invasion, trophoblast proliferation, angiogenesis, glucose metabolism, and immunological signaling (Salomon et al., 2014b). The mission to these exosomes is assigned by the placenta. Evidential studies have praised the similarities between the placenta and cancer on the behalf of their mechanism for evasion of immune response utilizing exosomes, thus generating a fetal or tumor-sustaining environment (Holtan et al., 2009). Such similarities have puzzled the brilliant scientific mind for ages, hence it is fascinating to connect and observe the underlined mechanisms. This review emphasizes how these factors (immune-exosomes) interact with the immune cells to modify their functions and affect their metabolic rates so as to yield a balanced pro- and anti-inflammatory milieu for successful fetal development and timely parturition. A well-sustained fetal development and timely parturition are based on a well-regulated immune clock implicating a pro-inflammatory milieu in the first and third trimester along with a skewed but required anti-inflammatory milieu in the second trimester (Dekel et al., 2010). Alterations of this stringent immune clock result in pregnancy complications like pre-eclampsia (PE), gestational diabetes (GDM), and preterm birth (PTB) (Erlebacher et al., 2007; Schonkeren et al., 2011; Han et al., 2015). The mass manipulation of the immune system by cancer cells via exosomes, for their survival, can be instigated for the ideas in mending the immune perturbations resulting in pregnancy complications. Therefore, we attempt to explore the role of the immune-exosomes in cancer and pregnancy focusing on taking lessons from the trail followed by cancer-derived immune-exosomes, which can help in the development of future therapeutics for pregnancy complications.

Further, we envisage that bringing about modification at the immune level with the use of exosomes as immunomodulatory effectors may prove as therapeutic tools, as have been studied in building up a strong tolerogenic niche for cancer survival.

### Conjunction of Bio-Molecules in a Healthy Pregnancy

Hormones, miRNAs and metabolites impact various immune cells and alter their lineages resulting in modification of their effector functions. This causes disbalance of pro- and anti-inflammatory milieu leading to adverse pregnancy outcomes.

### Hormones: The Catalysts of Pregnancy

**FIGURE 1:**
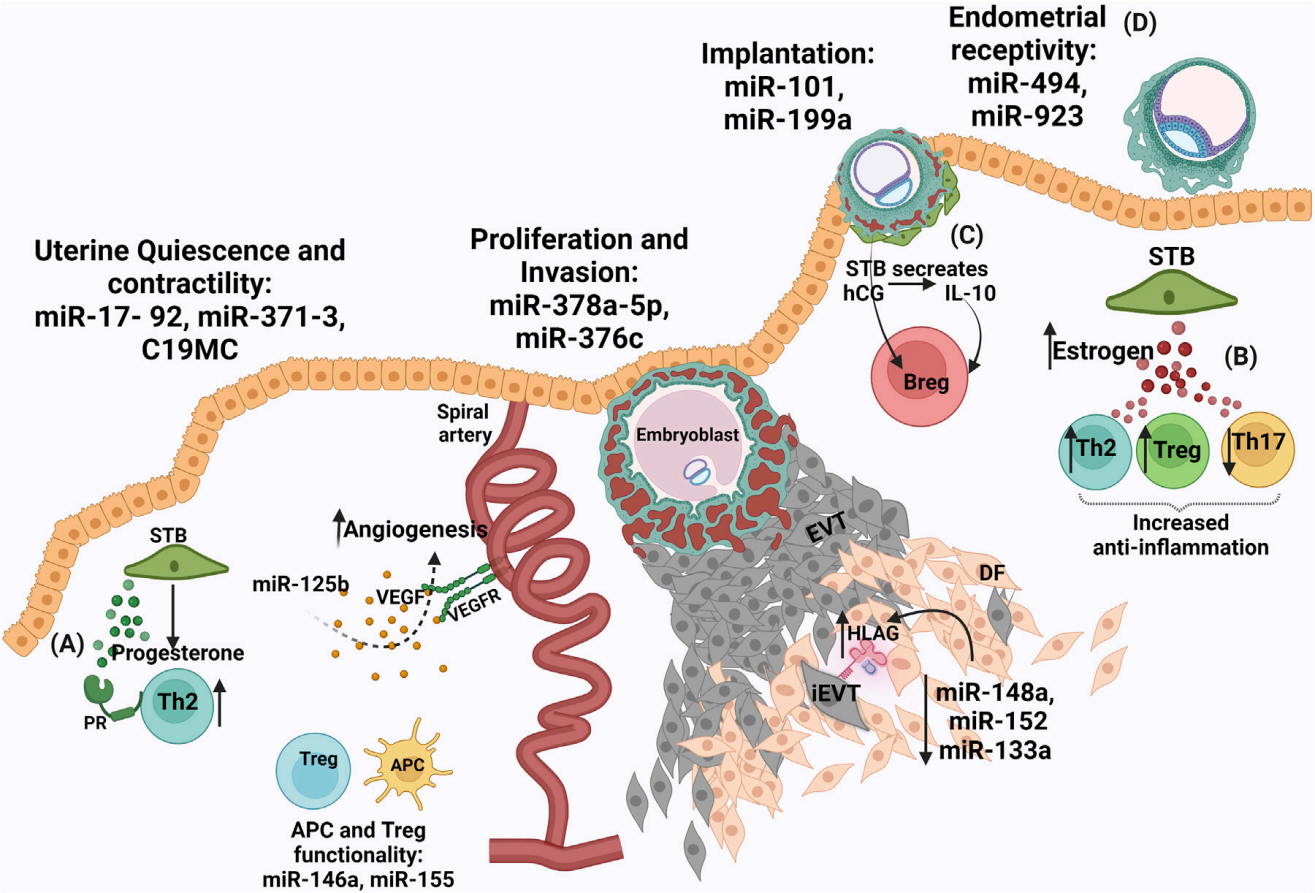
Conjunction of biomolecules in healthy pregnancy **(A)** Progesterone from syncytiotrophoblasts (STB) causes Treg expansion to form a tolerogenic zone **(B)** Increased estrogen levels from STB aids an anti-inflammatory response **(C)** Human chorionic gonadotrophin hormone (hCG) released from STB induces interleukin-10 (IL-10) which causes expansion of regulatory B cells and an assured immune environment **(D)** miRNAs are involved during placentation for endometrial receptivity: miR-30 family, miR-494, miR-923, implantation: miR-101 and miR-199a, proliferation and invasion: miR-378a-5p and miR-376c, uterine quiescence and contractility: miR-17- 92, miR-371–3, C19MC, APC and Treg functionality. miR0146a and miR-155 are involved in both pro- and anti-angiogenic functions. Reduced expression of miR-148a, miR-152, miR-133a in the extravillous trophoblasts (EVTs) ensures high levels of HLA-G to aid in a conducive environment by regulating NK cell cytotoxicity.

Progesterone, in most mammals, is essential for successful implantation and maintenance of gestation. Progesterone acts through its two nuclear progesterone receptor (PR) isoforms, PRA and PRB (Li et al., 2004; Mulac-Jericevic and Conneely, 2004). The A isoform is responsible for fertility in mice and B is involved in the development of the mammary gland (Mulac-Jericevic et al., 2000; Mulac-Jericevic et al., 2003; Conneely et al., 2003). It also lays a tolerant immunological environment in the endometrium, to shield the fetus expressing paternal antigens from the maternal immune attack responses. In peripheral blood, both PR isoforms are expressed on NK cells (Arruvito et al., 2008). During a healthy pregnancy, a significantly upregulated expression (approx. 97%) of PRs on γδ-TCR positive T-cells has been reported. However, in non-pregnant individuals the expression of PRs on γδ-TCR positive T-cells was reported to be as low as 14% (Polgar et al., 1999). Interestingly, the increased progesterone levels during a healthy pregnancy have been reported to induce progesterone-induced blocking factor (PIBF), which suppresses NK cytotoxic activity in the decidua thus, aiding successful pregnancy (Kandzija et al., 2019). Progesterone is crucial as a mediator to induce the naïve T cells to differentiate into Th2-type cells and inhibit activities of T effector cells, especially Th1 (Piccinni et al., 1995) (Figure 1A). Lower levels of PR on peripheral blood lymphocytes and serum PIBF have been associated with women having recurrent miscarriages (RM) (Liang et al., 2021). Lymphocyte immunotherapy has shown an improvement in outcomes for RM and is reported to induce increased PR expression on maternal lymphocytes (Hudic et al., 2020). In preeclamptic rat models, administering PIBF displayed normalized Th1/Th2 ratio, it suppressed inflammation, adjusted blood pressure to normal, and prevented fetal growth restriction. PIBF is detectable in the serum after 14 days of embryo transfer *in vitro* fertilization (IVF) patients PIBF concentration in serum increase with gestational age in normal pregnancy. However, a lower-than-normal threshold can help predict spontaneous pregnancy termination (Lim et al., 2020). Dydrogesterone treatment on peripheral blood mononuclear cells (PBMCs) isolated from women with a history of unexplained RSM induces Th2 responses by elevating IL-4 and IL-6 while suppressing Th1/Th-17 cytokines such as IFN-γ (Interferon-Gamma), TNF-α, and IL-17. Dydrogesterone treatment to women at risk of preterm delivery also resulted in increased PIBF production, IL-10 concentrations, and lower concentrations of IFN-γ (Lim et al., 2020).

Estrogens are extensively produced by the fetoplacental unit and required in maintaining pregnancy as well as for fetal maturation. The receptors for estrogens, similar to progesterone receptors are of two types, estrogen receptors (ER) -alpha (α) and -beta (β) (Bukovsky et al., 2003). These receptors are differentially expressed on subsets of immune cells such as lymphocytes, macrophages (MØ), and dendritic cells (DCs) (Kadel and Kovats, 2018). Increased expression of estrogen is associated with healthy pregnancies (Levitz and Young, 1977). Estrogen expression by the placenta raises the level of the hormone in circulation during gestation. Elevated expression levels of ER-alpha are found on T cells whereas, ER-beta elevated expression is reported on B-cells (Phiel et al., 2005). Estrogen exposed immune cells executes paired responses such that it can enhance NK cell cytotoxicity and interferon-gamma (IFN-γ) production but can also suppress granzyme B and FasL to increase and reduce inflammation, respectively (Hao et al., 2007). A dose-dependent effect of estrogen is observed on monocytes, where lower levels of estrogen result in an increase of pro-inflammatory interleukins (IL) IL-1, IL-6, and TNF- α and the higher level of estrogen reduces these pro-inflammatory cytokines (Bouman et al., 2005). In adaptive immunity, a higher concentration of estrogen promotes Th2 responses, expands regulatory T (Tregs) cells, and causes suppression of Th-17 in mice (Figure 1B) (Arruvito et al., 2007; Mao et al., 2010). Estrogen also aids angiogenesis by upregulating VEGF and VEGFR1, during normal pregnancy (Storment et al., 2000). Lower levels of estrogen in this aspect result in dysfunctional angiogenesis contributing to PE (Cantonwine et al., 2019). Short intramuscular-administration of estrogen in pre-eclamptic women reduces mean arterial blood pressure (Babic et al., 2018). Genistein, a phytoestrogen that works by binding G-protein ER (GPER) is used to treat PE. Lower levels of estrogen resulted in insulin resistance and thus are also associated with GDM pregnancies (Fernandez et al., 2016).

hCG, driven by the endocrine and immune system, induces maternal immune cells via lectin-glycan interactions to promote the attachment of the embryo to aid its invasion. Signals from embryo to endometrial immune environment lay a healthy embryo–endometrial relationship, producing pregnancy-induced immune tolerance in favor of the fetus. This stability deciphers the acceptance of the embryo for successful implantation (Schumacher et al., 2009; Schumacher et al., 2013; Schumacher and Zenclussen, 2019). hCG, via hCG receptors, stimulates IL-10 which is shown to increase CD19 + CD24 (high+) CD27 ^+^ regulatory B-cells population (Figure 1C). These regulatory B-cells enhance the positive effects of the immune environment in pregnancy (Rolle et al., 2013). In baboon endometrial stromal cells and glycodelin in the glandular epithelium, hCG was found to be directly involved in the induction of α-smooth muscle actin (SMA) expression. This suggests that the primate blastocyst, prior to implantation mediate changes in the uterine environment. Concomitant signals between the embryo and maternal endometrium form a cross-talk, which directs the event of successful embryo implantation (Fazleabas et al., 1999). A study demonstrated that hCG hormone is encapsulated in placental-derived exosomes and amnion-derived exosomes forming a no-contact bridge between maternal and embryo thus, providing distal effects. hCG from chorionic trophoblast cells is found to be involved in DC differentiation, maturation, and function regulation at the maternal-fetal interface (Fitzgerald et al., 2018).

### Pregnancy Involved miRNAs

Shown to be multiplayer, miRNAs are involved in inhibition of mRNA and promotion of translation (Taganov et al., 2007; Vasudevan et al., 2007; Olsen and Ambros, 1999). In humans, endometrial receptivity associated miRNAs are miR-30 family, miR-494, and miR-923, whereas, miR-101 and miR-199a aid the embryo implantation (Altmäe et al., 2013; Chakrabarty et al., 2007). miRNAs regulating placental functions like uterine quiescence and contractility are miR-17- 92, miR-371-3, chromosome 19 miRNA cluster (C19MC), miR-200 whereas, miR-378a-5p and miR-376c are involved in proliferation and invasion of trophoblast (Figure 1D) (Renthal et al., 2010). The myeloid cell differentiation has been reported to be regulated by miR-20a, miR-17-5p, and miR-106a. Clusters namely, C19MC, miR-371-3 (both located on chromosome 19), and C14MC (located on chromosome 14) are reported, out of which the C19MC is the most extensively researched (Flor et al., 2012). C19MC is expressed in trophoblast and placenta-derived stromal cells. miRNAs from this cluster are expressed in human embryonic stem cells and function in cell proliferation, invasion, and differentiation processes. C19MC expression is recorded in extravillous trophoblasts (EVTs) and several malignancies. Several miRNAs are involved in both pro- and anti-angiogenic functions (Donker et al., 2012). Members of the miRNA-17-92 cluster (miR-17, miR -18a, miR -19a, miR -19b, miR-20a, and miR -92a) have been shown to have anti-angiogenic effects on the endothelial cell *in vitro*, and inhibition of these leads towards pro-angiogenesis (Doebele et al., 2010). This regulation potential towards angiogenesis by miRNAs is exploited by cancer cells (Alpini et al., 2011). miRNAs are also involved in generating tolerance, such that HLA-G expressed mainly by the EVTs of the placenta could be downregulated by miRNA (miR-148a, and miR-152) binding to its 39-untranslated region thus, masking trophoblast antigenicity and shielding it from the attack of NK cells (Manaster et al., 2012). Favorably, the expression of these miRNAs have been reported to be expressed at low levels in the placenta, thus aiding the higher expression of HLA-G to create a tolerogenic realm. Modulating the immune cells, miR-155 is required for DC differentiation and DC endocytic capacity. 109 miRNAs are reported to influence macrophage (MØ) differentiation and exhibit both pro-inflammatory and anti-inflammatory phenotypes (Ferretti and La Cava, 2014). miR-146a, miR-155, and miR-223 miRNAs are involved in Treg cell differentiation. miR-17–92, a polycistronic miRNA mediates the regulation and differentiation of antigen-specific IL10-producing natural Tregs (Tregs) (de Kouchkovsky et al., 2013; Herberth et al., 2014). The maternal blood isolated at the 34th week of gestation and umbilical cord blood isolated at the time of birth had a higher miR-223, expression which was correlated with the lower number of Treg cells implying the increase in inflammation required for parturition. miR-146a enhances the suppressive capacity of Treg cells and in turn, limits Th1 responses (Zhou et al., 2015). miR-146a regulates TLR signaling and produces cytokines by decreasing the inflammatory response. However, decreased expression of miR-146a-5p was present in decidual tissue from patients with recurrent spontaneous abortions (Zhao et al., 2018a). *In-vitro* culturing of bovine embryos, revealed an increase expression of miR-25, miR-302c, miR-196a2, and miR-181a in embryos that demonstrated ceased development from morula to blastocyst stage, as compared to the embryos that successfully attained blastocyst stage. Thus, indicating a correlation between miRNA expression pattern and embryo development (Kropp et al., 2014). A study concluded that miR-34 is involved in cervical remodelling in normal labor whereas (Hassan et al., 2010), mir-223-3p is associated with preterm labor regulating the immune system. In preterm labor, mir-223-3p regulates inflammasome activity and MØ activation via NLRP3 and Pknox1 thus, regulating IL-1beta production (Bauernfeind et al., 2012; Haneklaus et al., 2012). miRNAs are unstable species thus, are encapsulated in exosomes to increase their stability and provide a targeted delivery. For embryo implantation miR-17, miR-106a and miR-200c are the most abundant miRNAs in placental exosomes (Yang et al., 2011; Ng et al., 2013). Exosomal C19MC family provides anti-viral responses by executing autophagy and thus may protect developing fetus from infections (Dumont et al., 2017).

### Immune Metabolome

A healthy pregnancy requires degradation of stored energy to facilitate fetal development and achieve timely parturition, thus causing a shift of an anabolic state in the first and second trimester to a catabolic state in the third trimester. These shifts primarily regulate the physiological immune responses in normal pregnancy whereas, alteration in these can lead to pregnancy complications.

### NK Cells

mTOR signaling-dependent regulation of glycolysis and mitochondrial functions are enhanced and most importantly studied in NK cell activation. In response to IL-2/IL-12/IL-5, the NK cells are activated, which leads to upregulation of nutrient receptors like CD71 and CD98 causing increased expression of GLUT-1 in an mTOR-dependent manner. This energy is required by NK cells to interact with villous trophoblasts and produce required responses for trophoblast invasion, proliferation, and tolerance (Jabrane-Ferrat, 2019). This provided the nutrition and energy which are essential at the initial stage of pregnancy (Donnelly et al., 2014; Slattery et al., 2021).

### Macrophages

Differentiated phenotypes of MØ have varied glycometabolism pathways. Pro-inflammatory type-1 macrophage (M1) provide spontaneous responses against invading pathogens inside the body receiving their power by anaerobic glycolysis. However, anti-inflammatory M2 responses are long-lasting and generated by mitochondrial oxidative phosphorylation (Van den Bossche et al., 2016). In response to lipopolysaccharide (LPS) and IFN-γ exposures, the MØ mitochondrial oxidative phosphorylation is downregulated, which triggers a shift towards type-1 macrophage (M1) polarization by anaerobic glycolysis and pentose phosphate pathway (PPP). Adding, hexokinases and GLUT1 are positively regulated by accumulated TCA cycle metabolites and increased HIF-1α (Tannahill et al., 2013). M1 are responsible for regulating the trophoblast invasion and proliferation by providing optimal inflammation during the early phase of pregnancy. However, prolonged dysfunction of mitochondrial oxidative phosphorylation is responsible for the generation of pro-inflammatory conditions like PE, gestational diabetes mellitus (GDM), and preterm birth (PTB). Thus, researchers have targeted the metabolic programming for the reversal of M1 to M2 polarization for therapeutic purposes. For instance, a study showed reconstruction of dysregulated mitochondrial oxidative phosphorylation by inhibiting iNOS thereby, reverting polarized M1 into M2 ultimately reducing the inflammation (Van den Bossche et al., 2016).

### Dendritic Cells

The activation of DCs and stimulation of DCs via LPS leads to inactivation of mitochondrial oxidative phosphorylation and thus a prompt response is generated to increase glycolysis rate for increasing the ATP production (Brombacher and Everts, 2020). The inhibition of mitochondrial oxidative phosphorylation occurs due to endogenous synthesis of NO by iNOS enzyme and stabilized HIF-1α via mTOR signalling. Amino acids like leucine, glutamine, (required for mTORC1 activity), and arginine (fuel for NO production) also affect mTOR signalling (Everts et al., 2012; Lawless et al., 2017). When DCs interact with T cells for antigen transfer, uptake of glucose and amino acids increases, yielding nutrient competitive surrounding and this competition cause prolonged T cell responses. However, these prolonged T cell responses are regulated on the type of T cell subset demand during the course of pregnancy. Extended inflammatory Th cell responses have been associated with pregnancy complications like GDM (Lawless et al., 2017).

### T Cells

Stimulatory responses by T cells are produced via switching between glucose and lipid metabolism, whereas the quiescent state of T cells is provided via oxidative phosphorylation (Warburg, 1956). T cell proliferation consumes a high concentration of ATP which is produced via conversion of pyruvate into lactate during glycolysis. Thus, producing essential bio-macromolecules for executing physiological processes of a cell such as growth and division (Pearce et al., 2013). Moreover, T cell stimulation requires increased absorption, this happens by the interaction between its co-stimulatory molecule (CD28) and TCR on APC. This interaction increases GLUT1 expressions via mTOR signalling resulting in increased glucose uptake by respective cells resulting in the execution of their effector responses (Macintyre et al., 2014). In the T cell subset, Th1, Th2, and Th17 closely rely on mitochondrial metabolism with Th17 being the fastest and longest consumer of glucose in a HIF-1α dependent manner (Dang et al., 2011). In addition, Treg cells have multiple metabolic pathways such as glycolysis, lipid oxidation, and oxidative phosphorylation regulating their responses. A regulated balance between these metabolic pathways for pro and anti-inflammatory cells exists (Michalek et al., 2011) however, mitochondrial metabolism could be targeted to decrease inflammatory T-cell responses. Similarly, to receive Treg prominent responses, its respective metabolic pathways could be targeted in creating therapeutics for chronic inflammation-associated pregnancies. The transport of these metabolic signals to the target cell could be via simple diffusion or carrier-mediated (Hardy et al., 2009; Weiler et al., 2017). During pregnancy, the role of exosomes in carrying immuno-metabolic signals to the target cell is still unclear and requires more attention. Although, the communication in the immune cells during pregnancy is crucial for fetal protection.

### Immune System in Pregnancy: Simply Complex

During the first trimester of pregnancy MØ, DCs and NK cells infiltrate the decidual tissue surrounding the invading trophoblast cells (Ashkar et al., 2000; Shimada et al., 2006). The events of implantation and placentation along the first and early second trimester of pregnancy display a close resemblance to “an open wound” which requires strong inflammatory responses (Dekel et al., 2010). In the first trimester, the human decidua has been reported to demonstrate a high number of immune cells, such as NK cells (70%), MØ (20–25%), DCs (1.7%), T lymphocytes (3–10%) with relatively lower expression of B cells in the decidua (Bulmer et al., 1988; King et al., 1997; Aluvihare et al., 2004; Zenclussen, 2005; Wicherek et al., 2009; Benner et al., 2020).

### Innate Immune Cell Cross-Talk During Pregnancy

**FIGURE 2:**
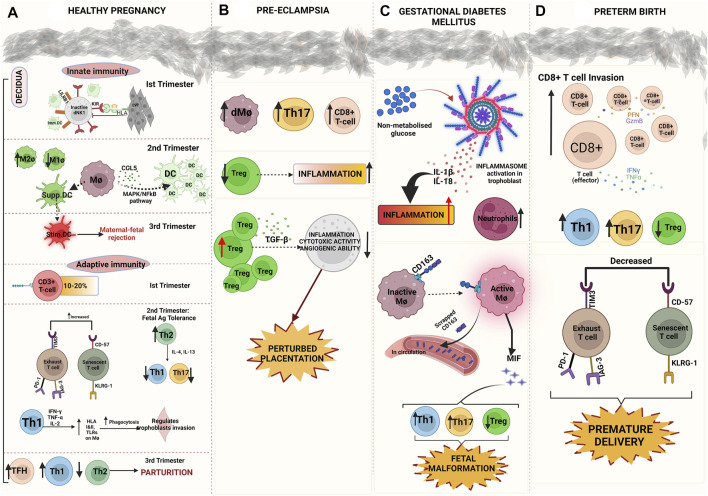
Immununological changes in pregnancy across gestation and in complications. **(A)** Healthy pregnancy: Innate immune responses: HLA disparity on extravillous trophoblasts (EVTs) causes NK suppression (first Trimester), increased M2Ø expression, production of DCs recruiting chemokine CCL5, accumulation of immature immunosuppressive DCs in decidua (second Trimester) for progression of pregnancy, differentiation of immunosuppressive DCs into stimulatory DCs contributing to parturition (third Trimester). Adaptive immune responses: 10–20% CD3^+^ Tcells exists in first Trimester, increased exhausted and senescent T cell population, increased Th2, decreased Th1 and Th17, pro-inflammatory cytokines (IFN-γ, TNF-α, IL-2) stimulate MØ to regulate trophoblasts invasion by phagocytosis (second Trimester), Increased expression of Th1, TFh, and decreased expression of Th2 promotes parturition as the gestation ends (third Trimester) **(B)** Immune dysregulation in pre-eclampsia (PE)- Increased expression of decidual MØ, T helper 17 cells, CD8^+^ cytotoxic T cells, increased suppression of regulatory T cells (Tregs) all these events lead to inflammation. Hyper-activation of Tregs causes decreased cytotoxic and angiogenic responses from NK cells leading to perturbed placentation causing PE **(C)** Gestational Diabetes mellitus (GDM) Increased levels of non-metabolized glucose induces inflammasome activation causing cytokine storm (IL-1β, IL-18) and increased neutrophilia leading to hyper-inflammation (MØ) activation causes cleavage of CD163 surface marker and its release into circulation, MØ releases MØ migration inhibitory factor (MIF) that further stimulates Th1 and Th17 promoting inflammation in GDM pathogenesis **(D)** Preterm birth (PTB) Increased CD8^+^ cytotoxic T cells infiltrate the decidua, increased Th1, Th17, and decreased Treg expression promote inflammation. Decreased expression of exhausted and senescent T cells are involved in PTB pathogenesis.

DC-mediated NK cells activation induces innate immune response whereas, NK-mediated DC editing and maturation activate adaptive immune response (Ferlazzo and Morandi, 2014). DCs and NK cells have been successful in establishing a reciprocal cross-talk in the decidual tissue across the pregnancy, in a direct or in an indirect manner by either cell-cell contact or by cytokine secretions, respectively (Figure 2A).

It was shown that over 60% immature DCs (imDCs) in the decidua were in close vicinity of NK cells (Kämmerer et al., 2003). Displaying a pregnancy-specific interaction, this clustering phenomenon between DCs and NK cells have been observed at the maternal-fetal interface (Tirado-González et al., 2012). The progression towards the second trimester occurs when IL-1β and TNF-α induce MØ and produce DC-recruiting chemokines through the MAPK and NFκB pathways (Li et al., 2011). CCL2 is the main chemoattractant for MØ and CCL5 is the main chemoattractant for immature DCs (imDCs). This results in the accumulation of MØ and DCs in decidual tissue (Figure 2A). Overexpression of anti-inflammatory genes, such as TGF-β is also reported (Dekel et al., 2014). In the second trimester, decidual MØ differentiates into immunosuppressive DC-like cells. There is an interesting shift of such immunosuppressive DC-like cells to immunostimulatory DC-like cells in the third trimester of pregnancy. This demonstrated a conclusive shift of maternal-fetal immunotolerance to maternal-fetal immune-rejection (Figure 2A) (Wang et al., 2016). Decidual MØ is believed to initiate childbirth through increased expression of inflammatory mediators to promote uterine contraction, parturition, and placental detachment (Bollapragada et al., 2009). In humans and rats, the MØ population was found to be increased in the decidua and also recruited to the cervix during ripening prior to the parturition (Päzolt and Henkert, 1990; Sakamoto et al., 2005). MØ subtypes work altogether to execute an optimal trophoblast invasion and spiral artery remodelling during healthy pregnancy. This occurs to meet the nutritional and respiration demands of the growing fetus. During the invasion of EVTs into the uterine stroma, a combinational profile of M1/M2 is established (Jaiswal et al., 2012). For the sustenance of the uterus and hence to avoid its rejection, a shift towards a predominantly M2 phenotype is observed (Figure 2A) (Mor et al., 2011).

On the basis of CD-11c expression, MØ are classified into two distinct groups in the decidual tissue during the first trimester (Houser et al., 2011). CD11c_high_ and CD11c_low_ secrete pro-and anti-inflammatory cytokines thereby helping in maintaining immune homeostasis during the first trimester while retaining defense against invading pathogens at the maternal-fetal interface (Houser et al., 2011). Conversely, gene expression profiling and surface marker phenotyping demonstrate that the term MØ resembles M2 skewed cells (Gustafsson et al., 2008; Repnik et al., 2008; Xu et al., 2016). Term MØ in decidua exhibit an immunomodulatory property with low expression of CD80/CD86 and produce major volumes of the immunosuppressive cytokine IL-10 (Heikkinen et al., 2003). Along with IL-10, trophoblast-derived macrophage colony-stimulating factor (M-CSF) in maternal monocytes have been proven to induce this M2 regulatory phenotype (Svensson et al., 2011). Soluble HLAG5 has been found to induce MØ by polarizing them to bear immunomodulatory phenotype exhibiting increased numbers of activated MØ (CD163 high) but decreased CD86 expression (Lee et al., 2015). Interestingly, in placental MØ pro-M2 genes like CCL2, CCL13, CCL14, and CD209 are hypomethylated to induce an M2-like phenotype and M1 phenotype is repressed by the hypermethylation of genes such as TLR-9, IL1B, IL-12 receptor β-2, and CD48 (Kim et al., 2012). To regulate angiogenesis in the feto-placental vasculature, a hallmark of organogenesis, placental MØ secretes VEGF and fibroblast growth factors (FGFs) like FGF2 (Demir et al., 2004; Loegl et al., 2016). Phenotypically characterized as M2-like, placental MØ can induce a pro-inflammatory response when activated via TLRs (Young et al., 2015; Thomas et al., 2021) and function to impart host defense within the placenta thus, triggering the local inflammation required for the initial development of the placenta (Young et al., 2015).

### Adaptive Immune Responses in Pregnancy

T cells constitute 45–60% of the total leukocytes in the endometrium in the early proliferative phase but the percentage decreases at the time of pre-conception creating a conducive environment for implantation (Gomez-Lopez et al., 2010; Bulmer et al., 1991). CD3^+^ T lymphocytes are present around 10–20% (Figure 2A) of the endometrial stromal leukocytes in the first trimester. Among the entire T cell population, CD4^+^ T cells (30–45%) and CD8^+^ T cells (45–75%) along with Th2 and Th17 cells accounting for 5 and 2% of CD4^+^ T cells, respectively (Bulmer et al., 1991; Nancy and Erlebacher, 2014). Nearly 5–30% of CD4^+^ T cells are found to be Th1 (CCR4-CXCR3+CCR6−) cells and nearly 5% CD25hi FOXP3+ Treg cells are CD4^+^ T cells (Nancy and Erlebacher, 2014).

In the early phase of pregnancy, the inflammatory priming of PBMCs occurs at the implantation site (Germain et al., 2007). Circulating syncytiotrophoblast’s microparticle (STBM) stimulates the production of various inflammatory cytokines, like IL-12, TNF-α along with mild-level of IL-18, from monocytes leading to the establishment of mild systemic inflammation (Sargent et al., 2006). On the surface of CD4^+^ T cells, chemokine receptor expressions (especially CCR molecules) determine their trafficking patterns which include the recognition of target tissue, timing, and signals to receive (Knieke et al., 2012). To keep track of the number and movement of trophoblast and prevent excessive trophoblast invasion, Th1 cells secrete cytokines IL- 2, TNF- α, and IFN-g (Figure 2A) (Torchinsky et al., 2003). TNF-α has been reported to act as a protector of the fetoplacental unit and regulates trophoblast invasion, by altering trophoblast cell adhesion to laminin and inhibiting the mobility of trophoblast cells studied through *in vitro* approaches (Todt et al., 1996). TNF-α hikes the trophoblast-derived plasminogen activator inhibitor-1 (PAI-1) levels and neutralizes the invasive capacity of trophoblasts (Bauer et al., 2004; Renaud et al., 2005). It has been stated that IFN-γ is involved in vascular remodelling during the peri-implantation phase and IFN-γ mRNA expression has been visualized at the implantation sites of healthy pregnant women and the murine model (Delassus et al., 1994; Jokhi et al., 1994). IFN-γ has a critical role of regulating EVT invasion, by increasing apoptosis of EVT and/or decreasing protease activity. Contrary to the physiological roles of IFN-γ, it impels pro-inflammatory functions as it increases expression of HLA class I and II antigen and TLR in innate immune cells (Podaný et al., 1975) which in turn promotes various functions like isotype commutation, chemokine secretion, (MØ) activation, and increased phagocytosis (Raphael et al., 2015). Pregnant women in the third trimester when compared to the non-pregnant counterparts have a higher percentage of peripheral blood follicular T helper cells (Tfh), despite co-expressing markers, including programmed death (PD)-1, ICOS, or CXCR3. Pregnant women also reveal a notably higher percentage of CXCR3C Tfh cells than non-pregnant women, which may produce IL-6, IL-10, and IL-21, and particularly, PD-1/CXCR3 (Monteiro et al., 2017). Th9 cells, a subpopulation of Th2 cells differ by altered phenotypical and functional aspects, which subjects to PPARγ involved in fatty acid storage and glucose metabolism (Micossé et al., 2019). In the presence of TGF-β, Th-17 cells produce IL-9 which have an inflammation-inducing function. In mouse, IL-9 was reported to be present in the non-pregnant uterus. However, during pregnancy, high level of IL-9 remained in both the placenta and uterus pointing again to its role in local inflammatory immune responses which might pose a threat to the developing offspring (Habbeddine et al., 2014). IL-22 secreted by Th22 cells has been found to be relevant for physiologic immune regulation and pathologic allograft rejection, therefore could potentially harm the pregnancy (Jia and Wu, 2014). At the maternal-fetal junction, IL-22 promotes proliferation, reduces apoptosis of trophoblast cells, and positively affects their viability (Wang et al., 2013). IL-22 plays an important role in protecting trophoblast cells from pathogens and producing inflammatory immune responses following intrauterine infection (Graham et al., 2011; Dambaeva et al., 2018). IL-22 receptors (IL-22R) are located on placental villi, a subunit of IL-22R, IL-22R1, allows binding of IL-22 from dNK cells and decidual stromal cells (Dambaeva et al., 2018). The downstream IL-22/IL-22R1 pathway is said to be involved in the trophoblasts survival and maintenance of pregnancy. In a successful pregnancy, IL-22, Th17/Th2 and Th17/Th0 subsets were highly prevalent, and the mRNA expression of GATA-3, ROR-C, AHR, IL-4, IL-17A, and IL-22 were recorded at the site of implantation. However, mRNA expression of T-bet and IFN-γ was detected away from the site of implantation. Hence, for a successful pregnancy, the pertinent association of IL-22 and IL-4 production at the implantation site is proved (Logiodice et al., 2019).

### Immune Tolerance in Pregnancy

In healthy pregnancy, the earlier defined Th1/Th2 paradigm shifted to Th1/Th2/Th17/Treg paradigm when the advancement in the understanding of feto-maternal immune cross-talk for building a fetal alloantigen tolerogenic environment became clearer. The shift of pro-inflammatory milieu to anti-inflammatory milieu majorly occurs during the second trimester of pregnancy where fetal tolerance is at its maximum while at the end of the third trimester of pregnancy shows the generation of fetal rejecting environment to induce parturition (Chaouat and Voisin, 1979; Saito et al., 2010). In the early pregnancy development of fetal tolerant surroundings takes place when the maternal immune system encounters paternal antigens on the fetus, which causes phenotypic suppression of maternal immune cells. This suppression of immune cells is contributed from both fetal and maternal side. It has been reported that even fetal immune cells in response to maternal antigens cause inactivation of inflammation producing fetal immune cells and expansion of anti-inflammation producing fetal immune cells. In addition, the construction of fetal trophoblasts is in such a way that they escape maternal immune cell attack. The cytotrophoblasts, and STB along with STBM do not express any variety of HLA or NOD-like receptor family CARD domain containing 5 (NLRC5) (Tilburgs et al., 2017). Thus, during healthy pregnancy, the alloreactivity of CD3^+^CD4^+^ T helper cell is suppressed in the absence of HLA class I and II antigens on villous trophoblasts. In contrast to villous trophoblast, EVTs expressed HLA C, a classical MHC class I molecule, and a non-classical MHC class I molecules HLA E, F, and G and MHC transcriptional activators such as NLRP2 (Tilburgs et al., 2017; Tilburgs et al., 2010). At the maternal-fetal junction, HLA-C histo-incompatibility has been recorded to induce a tolerogenic microenvironment (Tilburgs et al., 2009). Prior to implantation, paternal antigen-specific Treg cells accumulate and increase in number in the uterus after implantation. Intriguing results from (Mohr et al., 2019) showed how seminal plasma initiates the expansion of Treg cells specific to paternal antigens imparting tolerance to paternal alloantigen (Shima et al., 2015; Robertson et al., 2009). As the pregnancy progresses, the cellular responses of innate and adaptive immunity work in collaboration to strengthen and extend fetal tolerance. DCs drives differentiation of naïve T cells into Th2 and Tregs in response to fetal antigen exposure. Increased Th2 response causes secretion of anti-inflammatory cytokines like IL-4, IL-5, IL-6, IL-10, IL-13, and TGF-β thereby decreasing the local inflammation. IL-4 and IL-13 work in a paracrine manner and represses Th1 and Th17 immunities, respectively, and brings forth allograft tolerance (Figure 2A) (Mitchell et al., 2017). Another subset of T cells like CD8^+^ Tc cells upon indirect recognition of fetal antigens, undertake the fate of clonal deletion (Erlebacher et al., 2007) whereas, CD4^+^CD25 + Fox3+ Treg expansion has been found to establish and maintain an allogeneic pregnancy in both mice and humans(Zenclussen et al., 2005). Treg cells play a crucial role in the production of paternal antigen-specific tolerance (Rowe et al., 2012). Another physiological phenomenon of inducing tolerance during pregnancy is T cell exhaustion and senescence which are known to occur because of excessive stimulation of T cells. This causes T cells to lose their proliferative and cytokine secreting properties however, the exact mechanism leading to this is still unknown. T cell exhaustion and senescence is characterized by increased surface expression of inhibitory receptors like PD-1, TIM-3, CTLA4, LAG-3 and CD57, KLRG-1, respectively (Figure 2A) (Sugita et al., 2013). PD-1/PD-L1 (CD274) axis engages in the suppression of autoreactive immune effectors and to achieve T cell homeostasis. Through negative costimulatory interactions, the PD1/PD-L1 pathway can also suppress Th22 and Th9 cells (Wang et al., 2020a). Primarily, identified as a Th1-specific receptor, Tim-3 is present on the surface of the cell. These domains engage galectin-9 (Gal-9) to transduce an apoptotic signal which ultimately results in inhibition of Th1 responses (Zhu et al., 2005; Miyanishi et al., 2007). The interaction of Tim-3 and its ligand Gal-9, causes intracellular calcium influx which commence the supersession of Th1 and Th17 cells (Seki et al., 2008; Oomizu et al., 2012). Conversely, Tim-3 enhances Th2 immunity at the maternal-fetal junction thereby safeguarding the decidual stromal cells from inflammatory damages and apoptosis mediated by TLR (Wang et al., 2015a; Wu et al., 2015). Therefore, Tim-3 signalling during pregnancy may operate as a self-control mechanism in TLR-triggered inflammation (Wang et al., 2015a). CD-57 expression is indicative of shortened telomere inside the cell implying that the cell has lost the ability to proliferate conferring a suppressed state of immune cell which is required for preventing fetal rejection (Slutsky et al., 2019). Later in pregnancy, paternal antigen-specific tolerance disappears post-delivery which is earlier present during pregnancy (Rowe et al., 2012). In a study, cytokine analysis of serum from pregnant women revealed the increased levels of IL-1b, IL-6, IL-8, IL-12p70, L13, IL-15, IP-10, and FLT3-ligand in relation to gestational weeks while, serum IFN alpha-2, IL-1RA, IL-3, IL-9, IL-12p40, and soluble CD40L levels were increased with the advancement of the trimester (Holtan et al., 2015). As interpreted, the optimal increase in pro-inflammation in the third trimester of pregnancy is associated with the preparation for the healthy delivery.

### Immune Dysregulation Causing Pregnancy Complications

Immune tolerance built by various diverse cellular interactions is the cornerstone for successful gestation and healthy outcomes. The breakdown of this mechanism is proved to be one of the causes for the pathophysiology observed in adverse pregnancy outcomes. Various studies have been performed to understand the immune dysregulation in the context of pregnancy complications like PE, GDM, and PTB.

### Pre-Eclampsia

PE is indicated as a state of hypertension and proteinuria any time after 20 weeks of gestation and is categorized as early-onset PE (EOP) that presents before 34 weeks and late-onset PE (LOP) that initiates after 34 weeks of gestation. A hallmark of PE is a deficiency of EVT infiltration and spiral artery remodelling, which results in a placental microenvironment that is ischemic towards increasing oxidative stress (Cartwright et al., 2017). Hyper-activation of pro-inflammatory cells (M1, Th1, Th17, cytotoxic dNK cells) or hyper-activation of anti-inflammatory cells (M2, Th2, Treg, suppressive dNK cells) causes alterations in the process of placental formation leading to pre-eclampsia. M1 have been reported to have elevated levels than M2 in the decidua of patients with PE, with a total increase in the MØ numbers in PE patients when compared to healthy controls (Schonkeren et al., 2011). Uterine M1 by the action of TNF-α has been reported to inhibit trophoblast invasion and disrupt spiral artery remodelling (Renaud et al., 2005). Similarly, the cytotoxic capacity of CD8^+^ T cells has been involved in controlling trophoblast invasion. In a human study, CD3^+^ and CD8^+^ T cells were significantly increased in the maternal decidua of PE patients compared to normotensive controls, indicating that an inflammatory environment aids in the progression of the disease (Milosevic-Stevanovic et al., 2019). Higher Th17/Treg ratios in umbilical cord blood, peripheral blood, and decidua have been reported to be associated with preeclamptic women when compared to healthy pregnant and non-pregnant controls (Figure 2B) (Milosevic-Stevanovic et al., 2019). In addition, animal studies have shown that depletion of Tregs in early gestation results in the generation of an uncontrolled pro-inflammatory milieu that causes preeclampsia-like phenotype (Care et al., 2018). This is suggestive of an exacerbated pro-inflammatory response that disturbs the trophoblastic properties of migration, invasion, and proliferation thus causing PE. However, contradicting studies have also been reported to be involved in PE pathogenesis. Increased expression of cytotoxic CD8^+^ T cells in PE patients’ decidua basalis, has also been reported by few studies and is suggestive of their role in the pathophysiology of PE (Milosevic-Stevanovic et al., 2019). Moreover, the increased number of dNK cells, decidual Treg cells, and TGFβ-1 in pre-eclamptic women is connected with a profound notion that excess anti-inflammation or increased suppression of cytotoxic and angiogenic properties of dNK cells can also result in insufficient trophoblasts proliferation, migration, and invasion. Thus, indicating the need for a balanced spatio-temporal relationship between inflammation and anti-inflammation for adequate spiral artery remodeling (Figure 2B) (Zhang et al., 2019). Another important aspect of PE pathogenesis is increased obstructions in maternal blood flow during pre-eclampsia, due to which dNK cells cannot interact with trophoblast cells and with other decidual cells, thus are restrained in promoting an adequate trophoblast invasion, causing dysfunction in spiral artery remodeling in PE (Fraser et al., 2012). However, inconsistent results are found over the varied role of dNK cells in PE giving the explanation of geographical indications, that even the environmental factors have an impact in modulating the immune system (Valenzuela et al., 2012; Shashar et al., 2020; Steinthorsdottir et al., 2020).

### Gestational Diabetes Mellitus

Affecting 15% of pregnant mothers in developing countries GDM is a metabolic disorder which if left untreated may result in PTB due to hyperglycemia (Salomon et al., 2016). Hyperglycemia in GDM is associated with increased inflammation which occurs due to activation of inflammasomes in trophoblasts. The potent reason behind this activation of the inflammasome is excessive glucose which induces NLRP3 resulting in the generation of pro-inflammatory cytokine storms mainly IL-1β and IL-18 (Figure 2C) (Han et al., 2015; Corrêa-Silva et al., 2018). Excessive neutrophilia, high glycaemic levels, and increased homeostatic model assessment of insulin resistance are associated with GDM diagnosis as early as in the first trimester (Figure 2C) (Sun et al., 2020). The increased numbers of neutrophils are intended to be more reliable than leukocyte numbers i.e., the neutrophil to leukocyte ratio is used as an inflammatory marker for diagnosis of GDM in the second trimester. In addition, during the third trimester of pregnancy for GDM prediction a serum delta neutrophil index representing increased neutrophil numbers and inflammation is adopted (Sahin Uysal et al., 2020). The innate immune system contributes to increased inflammation in GDM via inflammatory signals secreting monocytes (Chandra et al., 2012). Monocyte/MØ activation has been proposed to be an early predictor of GDM in as early as 14–16 weeks of gestation. A hemoglobin-haptoglobin scavenger receptor CD163 (sCD163) is scraped out of MØ as an activation marker of these cells and this shedding is significantly increased in GDM women thus, the increased circulatory levels of CD163 from the placenta as well as from adipose tissue are reflective of GDM (Figure 2C) (Dige et al., 2014). Another study reveals elevated levels of CD163 + cells, IL-6, TNF-α, and TLR2 are associated with a pro-inflammatory milieu in GDM patients when compared to healthy pregnancies (Ueland et al., 2019; Bari et al., 2014). Another MØ secretory signal, a pro-inflammatory cytokine known as MØ migration inhibitory factor (MIF) which stimulates TH1 cells, induces IL-17 release, and increases TLR-4 expression on MØ is used for GDM prediction (Figure 2C) (Yilmaz et al., 2012). Moreover, GDM susceptibility has also been determined by specific genotypes associated with MIF (Aslani et al., 2011; Zhan et al., 2015). Decreased Treg numbers are associated with GDM prognosis, as shown in multiple studies where subsets of suppressive Tregs, CD4^+^CD127LOW+/CD25 + Tregs and CD45RA Tregs were evaluated during GDM pregnancies and represented a decline of anti-inflammatory function of Tregs as early as in the first trimester of GDM pregnancy (Schober et al., 2014). In addition, CD4^+^ CD25 and CD4^+^CD25 + FOXP3 cells numbers were decreased whereas, TNF-α, a pro-inflammatory cytokine expression by Tregs (CD4^+^CD25 + FOXP3+CD127-) were found to be significantly upregulated in women with GDM pregnancies compared to women with healthy pregnancies (Schober et al., 2014). Aggravated circulatory CD4^+^ and CD8^+^ T cells responses in GDM pregnancy contribute to GDM pro-inflammatory milieu with significantly higher expression of CD69 (T cell activation marker) in insulin-untreated cases and higher expression of HLA-DR in insulin-treated cases (Lobo et al., 2018). Thus, the above-mentioned studies project towards an extensive pro-inflammatory build-up in GDM patients. In addition, increased levels of circulating Th-17 cells, a higher Th17: Treg cells ratio, and Th1: Treg ratios have been associated with GDM pregnancies compared to uncomplicated pregnancies (Sheu et al., 2018; Zhao et al., 2020). Thus, in order to predict a pregnancy complication only studying Th1/Th2 imbalance is insufficient however, a more comprehensive understanding can be attained by taking the Th1/Th2/Th17/Treg paradigm into consideration.

### Preterm Birth

PTB is defined globally as any live birth that occurs before 37 weeks of gestation or less than 259 days. According to the world health organization (WHO), an estimated 15 million infants are born prematurely every year. One-fifth of those 15 million prematurely born infants across the world are, born in India PTB is stratified as spontaneous PTB with an intact membrane (sPTB-IM), induced PTB, preterm premature rupture of membrane (pPROM), and caregiver induced PTB. Among the PTB populations, the prevalence of sPTB is 40–45%, induced is 30–35% and pPROM is 25–30% (Goldenberg et al., 2008). The immunological status of an idiopathic PTB is more complicated than that of PE or GDM because of the absence of pathological cues. Whereas, the infection-induced PTB and labor are more frequently studied. Neutrophils are the phagocytic cells that reach predominantly at the infection site or site of injury thereafter recruiting other effector immune cells. Several rodent studies have reported that depletion of neutrophils prior to LPS administration could not delay the preterm labor however, it did help in reducing the IL-1 beta levels at the feto-maternal interface (Arenas-Hernandez et al., 2019; Gomez-Lopez et al., 2016) implicating an indirect role of neutrophils in creating an inflammatory milieu underlying PTB or pPROM. Histological evidence of PTB placentae has shown a more prominent invasion of CD8^+^ Tc cells indicating chorioamnionitis as similarly observed in cases of pPROM and fetal death (Figure 2D) (Galaz et al., 2020). Flow cytometric analysis of these cases revealed an influx of effector memory T cells, secreting high levels of perforins and granzymes at the feto-maternal interface in preterm labor (Arenas-Hernandez et al., 2019). The chorioamnionitis membranes in preterm placentae are infiltrated by the increased number of Th17 subtypes that release IL-17 at the maternal-fetal interface and also in amniotic fluid indicating a chronic inflammatory status (Figure 2D) (Ostojic et al., 2003; Wu et al., 2014; Lombardelli et al., 2016; Pinget et al., 2016). At the feto-maternal interface, the elevated expression of Th1 and Th17 related genes with declined FOXP3 expressions were associated with unexplained recurrent pregnancy loss and spontaneous abortion patients (Lee et al., 2011; Wu et al., 2016; Zhu et al., 2017). Invariant NK cells (iNKTs) are the bridges between innate and adaptive immunity, where they provide an intense immune activation by upregulating the signalling pathways responsible for Th1 and Th2 cytokine release (Miller et al., 2018). Studies have reported increased expression of iNKT in the first and third trimester of pregnancy thus, implying their roles during term labor (Wang et al., 2002; Boyson et al., 2002). Preterm murine studies have revealed an inverse relation of iNKT and Tregs at the feto-maternal interface (Gomez-Lopez et al., 2017). The expansion of iNKT cells was accompanied by increased Th17 and decreased Treg expression. Thus, inhibiting iNKT cells activation reduced the immune responses at feto-maternal interface, thus delaying preterm labour in mice (St Louis et al., 2016). Moreover, in humans increased expression of iNKT cells at the decidua were revealed in a transcriptomic analysis and immunophenotyping of lymphocytes in placentae of preterm cases when compared to control terms (St Louis et al., 2016). Given that iNKT cells are present at the murine maternal–fetal interface throughout pregnancy, other than the innate immune cells contributing to infection induced PTB, the adaptive immune cells also have important roles in PTB (Gomez-Lopez et al., 2017; St Louis et al., 2016). Exhausted and senescent T-cells are present at the maternal-fetal interface and help in regulating inflammation throughout gestation in a normal pregnancy. Chronic/repetitive antigen exposure on T cells can result in their functional loss which is identified by the expression of exhaustion markers such as TIM-3, PD-1, CTLA-4, and LAG-3. Whereas, T cell senescence is characterized by vanished proliferative ability along with the absence of these inhibitory markers and presence of senescent markers (increased CD57, KLRG-1 and decreased CD27 and CD28 (Wherry and Kurachi, 2015). In humans, CD4+T cells exhibiting effector memory phenotype showed upregulated expression of inhibitory marker PD-1 at the second trimester during normal pregnancy (Meggyes et al., 2020). During infectious preterm pregnancy, a decline in senescent CD4+/CD8+ T cell numbers and exhausted CD4^+^ T cell numbers have been reported at the feto-maternal interface (Slutsky et al., 2019). The existence of T cell subsets in the above-mentioned effector memory phenotypes concludes a pro-inflammatory milieu responsible for preterm labour leading to PTB (Figure 2D). Moreover, blocking the inhibitory markers using antibodies to PD-1, TIM-3 has been associated with increased rates of fetal loss and thus emphasizing the fact that balanced cellular exhaustion and senescence are required for the execution of a healthy pregnancy (Wang et al., 2015b). This was further supported by the observation that CD8+PD-1+TIM-3+ T cells were impaired in decidual tissues from women with miscarriage (Wang et al., 2015b; Slutsky et al., 2019). Another aspect contributing to the pregnancy complications as explained in PE and GDM also exists in PTB i.e., decrease in Tregs numbers. Immunophenotyping performed on the lymphocytes isolated from women undergoing preterm labor revealed that chorioamnionitis accompanied preterm labouring women at the time of delivery had significantly lower numbers of Tregs as compared to term labouring women (Xiong et al., 2010). Studies have revealed the existence of reduced Tregs at the feto-maternal interface in women with idiopathic preterm birth. In a mice model of endotoxin (LPS) induced PTB the depletion of Tregs in the third week of mice pregnancy resulted in PTB. The endotoxin-induced PTB was reversible by adoptive transfer of depleted Tregs from allogeneic mice, implying the importance of Tregs in delivering a full-term pregnancy (Gomez-Lopez et al., 2020). Moreover, human cellular studies are accompanied by cytokine studies, which represented a decrease in the levels of IL-10 an anti-inflammatory cytokine with each approaching trimester in PTB. Serum levels of IL-10 and IL-10 receptors in endometrial biopsy of women with preterm labor were also found to be lower when compared to women with normal labor (Pereira et al., 2016). However, the trigger behind the perturbed immune responses in idiopathic PTB still remains unclear and requires thorough investigations.

### Pla-Xosomes: Connecting Link Between Immune Clock and Pregnancy Complications

Ongoing research for identification of the one triggering factor responsible for bringing about perturbations of the immune system that lead to such pregnancy complications and adverse pregnancy outcomes is still unknown. However, of the multiple studies underway that are being investigated for identification of this trigger, one such investigation involves the study of extracellular vesicles also known as exosomes (EVs). Discovered almost 40 years ago in 1989 (Trams et al., 1981; Pan and Johnstone, 1983; Harding et al., 2013), the extracellular vesicles named exosomes were characterized later as lipid-bilayered-intraluminal microvesicles (ILVs) (30–150 nm) yielded by invagination of multivesicular bodies (MVBs) derived from endosomes during stress response or for cell-to-cell communication (Harding et al., 1984). Exosomes being the most biological residual structures with the least cytotoxicity are loaded with cargo in the form of RNAs (miRNAs) (Menon et al., 2019), proteins (cytokines) (Pillay et al., 2020), hormones (estrogen, progesterone (Fitzgerald et al., 2018), cDNAs, and metabolites making them chief molecules of cell-cell communication (Kurian and Modi, 2019). Since exosomes act as power shots of clues/factors for regulating the proximal and distal cellular responses, they are being studied to unravel the trail leading to the trigger of immune dysregulation in pregnancy complications. The involvement of exosomes in facilitating feto-maternal cross-talk during a successful pregnancy through reported literature on the cargo investigated at regular stages of gestation has led to a deeper understanding of these power shots as physiological modifiers through their action on the immune system of the pregnant mother. Exosomes act as messengers between the fetal and maternal tissues during pregnancy, delivering their payload to target cells towards making an incremental functional impact. They also have crucial roles e.g., in embryo implantation (Kurian and Modi, 2019), accelerating the glucose metabolism (James-Allan et al., 2020), and acting as a mediator for executing immune responses bring about either activation, suppression, or tolerance (Mincheva-Nilsson and Baranov, 2014a). In early pregnancy, exosomes produced by the placental cells (pla-xosomes) induce endothelial cells and vascular smooth muscle cells to promote angiogenesis (Salomon et al., 2014b). Apart from maintaining the conducive environment for the healthy growth of the developing fetus, the inflammatory signals required to initiate parturition at the last trimester of pregnancy are also provided by exosomes (Sheller-Miller et al., 2018).

### Exosomes Facilitate a Fetal Sustaining Environment During a Healthy Pregnancy

Exosomes from trophoblast cell lines have been reported to trigger the recruitment and differentiation of immune cells specifically monocytes. Placenta-derived exosomes (Pla-xosomes) concentration increases with each progressive gestation of a healthy pregnancy (Salomon et al., 2014a). Pla-xosomes can cause phenotypic changes in monocytes i.e., phagocytic classical monocytes (CD14++ CD16^+^) are transformed into intermediate monocytes (CD14 + CD16^+^) with enhanced migratory capabilities, and pro-inflammatory factors like IL-1beta, IL-6, serpin1, GM-CSF, M-CSF, and TNF-α are secreted (Al-ofi et al., 2012; Tagliani et al., 2011). These responses are essential to function in an optimal manner so as to provide regulated angiogenesis and invasion of trophoblast cells. Along with pregnancy, M1 polarization to M2 occurs to contribute to an anti-inflammatory phase for fetal survival (Figure 5B). This transition is caused by the presence of an immune checkpoint inhibitory molecule known as PDL-1 on the pla-xosomes (Petroff et al., 2003; Enninga et al., 2018). Effector responses of T cells have to be reduced in order to aid the successful growth of the fetus. Multiple mechanisms such as inhibition of T cell proliferation, T cells apoptosis, T regulatory expansion, and reduction of Tc cells occur so as to shield effector T cell responses (Figure 5B). The immune cells have been reported to express the FAS and TRAIL receptors. Interestingly pla-xosomes isolated from the placenta or that from blood biopsies express apoptotic molecules like FAS ligand and TRAIL, thus inducing apoptosis in Jurkat cells and activating PBMCs via their receptors in a dose-dependent manner (Stenqvist et al., 2013). In addition, pla-xosomes from maternal blood downregulate the expression of CD3 and JAK3 inhibiting T cell activation (CD4^+^ and CD8^+^) (Sabapatha et al., 2006). MHC class I chain-related (MIC) and UL-16 binding protein (ULBP) expression on pla-xosomes downregulates expression of NKG2D receptor on CD8^+^ T cells thus inhibiting their cytotoxic responses (Figure 5B) (Hedlund et al., 2009). Syncytin-2 an endogenous retroviral protein is expressed on pla-xosomes and has been reported to reduce Th1 cytokine secretion using PBMCs invitro culture causing immunosuppression (Figure 5B) (Lokossou et al., 2020). Although, pla-xosomes inhibit lymphocyte proliferation and induce regulatory/memory T cells differentiation in a similar manner the tumor-derived exosomes manipulate the immune cells by inhibiting immune cell attacks (Mikami et al., 2020; Yu et al., 2020). The induction of Tregs is crucial for the sustenance of the fetus during the second trimester of the pregnancy. EVs from BeWo cells showed expression of a 10 KDa heat shock protein which initiated the helper T-cell differentiation to Treg cells (Kovács et al., 2019). As described above the exosomes are potential mediators of cell-cell communication during a healthy pregnancy. The immune perturbations in pregnancy complications alter the cargo of exosomes and their numbers, which have been associated with perturbed pregnancies like pre-eclampsia, GDB, and PTB.

### Pla-Xosomes in Adverse Pregnancy Outcomes

#### Preeclampsia

**FIGURE 3:**
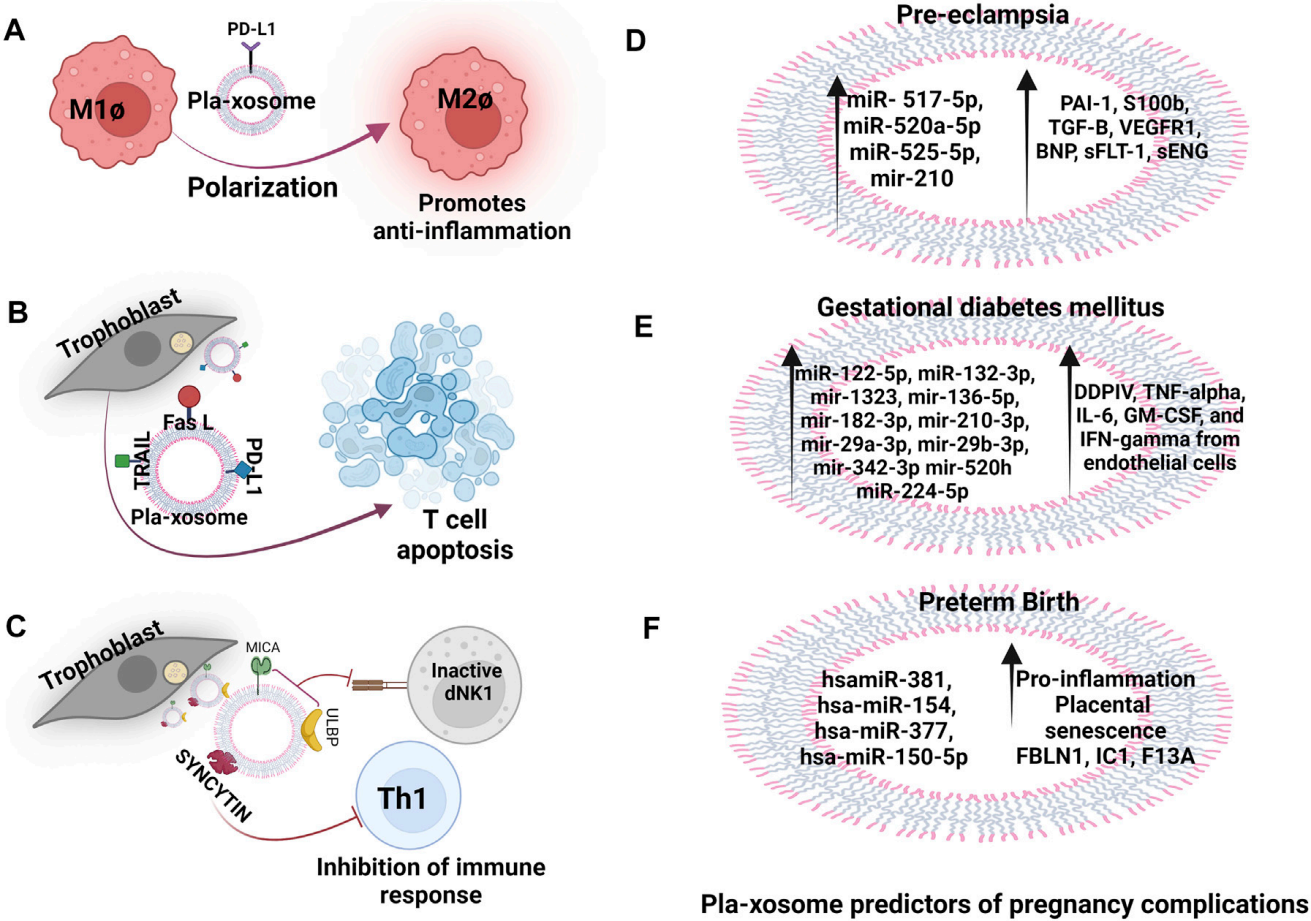
Pla-xosomes in heathy, complicated pregnancies and adverse outcomes. **(A)** Pla-xosome (PD-L1+) causes M1Ø to M2Ø polarization to increase anti-inflammation **(B)** Pla-xosomes (Fas L + TRAIL + PD-L1+) cause T cell apoptosis for regulated immunosuppression **(C)** Pla-xosomes (ULBP + MICA + Syncytin+) cause inhibition of innate and adaptive immune response to support restructuring of materno-fetal interface **(D)** Increased expression of pla-xosomal miR- 517-5p, miR-520a-5p, miR-525-5p, and miR- 210 and the proteins PAI-1, S100b, TGF-B, VEGFR1, BNP, sFLT-1, sENG as predictors **(E)** miR-122-5p, miR-132-3p, mir-1323, mir-136-5p, mir-182-3p, mir-210-3p, mir-29a-3p, mir-29b-3p, mir-342-3p and mir-520h along with proteins like DDPIV, GLP-1, tumor-necrosis factor-alpha (TNF-α), IL-6, GM-CSF, and IFN-gamma in GDM reported to be predictors of the disease **(F)** hsamiR-381, hsa-miR-154, hsa-miR-377, and hsa-miR-150-5p and proteins FBLN1, IC1, F13A are associated with preterm labour that causes increased placental senescence and inflammation.

Compared to a healthy pregnancy, the placental EVs from PE patients remain in circulation for longer. Pla-xosomes levels in pre-eclamptic pregnancies in the third trimester have been reported to be elevated in comparison to healthy control (Pillay et al., 2016). Exosomal cargo has been described as biomarkers for pre-eclampsia. In the C19MC miRNAs, a set of placental unique miRNAs (miR- 517-5p, miR-520a-5p, and miR-525-5p) measured in the first trimester were reported as a biomarker panel (AUC: region underneath the curve 0.719) for predicting the PE prognosis (Figure 3D) (Hromadnikova et al., 2019). Proteomic studies on pre-eclamptic maternal plasma-derived exosomes have revealed higher expression of peptidase inhibitor (PAI)-1, S100 calcium-binding protein (S100b), TGF-β, VEGFR1, and natriuretic peptide B(BNP) (Tan et al., 2017; Tan et al., 2014) compared to their healthy counterparts. Increase in sFLT-1 (soluble fms-like tyrosine kinase-1) and sENG (soluble endoglobin), the causative agents of PE are found to have upregulated expression in PE exosomes compared to controls (Figure 3D) (Chang et al., 2018). Providing the indications of PE pathology, a reduction of immune-suppressive markers like PD-L1 and syncytin 1 or 2 (regulates M1 polarization, T reg cell differentiation, and inhibits T cell activation respectively) on exosomal membranes have been reported in preeclamptic patients (Levine et al., 2020). RNA sequencing has revealed elevated enrichment of mir-210 in preeclamptic patients that downregulates potassium channel modulatory factor 1 and thus inhibits trophoblast invasion (Luo et al., 2014). In pregnant mice, exosomes derived from the plasma of PE patients can induce PE-like phenotypes in the mother as well as the fetus (Sheller-Miller et al., 2019). PE STBs derived-EVs induces the production of superoxide by neutrophils which have been thought to surge the neutrophil extracellular traps (NETs) formation and showed more interaction with monocytes, MØ, thus increasing the pathological inflammation (Gupta et al., 2006). Pla-xosomes carry the destined cargo to prepare the mother by modulating the physiological, structural, and immunological status towards the healthy development of the fetus.

#### Gestational Diabetes Mellitus

In humans, the PLAP content per exosome (PLAP ratio) is used to define the existence of placental exosomes in total exosomes. In GDM, this ratio has been found to be lower in comparison to normal pregnancy irrespective of the higher number of total and placental exosomes implying that there are alterations in the number of exosomes released by the placentae, increased non-placental exosomes secretion, or convergence of both (Salomon et al., 2016). Exosomes from the plasma of GDM patients also cause glucose intolerance, decreased glucose-induced insulin secretion, and poor insulin responsiveness (James-Allan et al., 2020). Exosomal miRNAs are extensively studied for the prediction of GDM in humans eg. miR-125a-3p, miR-99b-5p, miR-197-3p, miR-22-3p, and miR-224-5p are consistently detected in higher concentrations in the placenta, skeletal muscles, placental and total exosomes representing their metabolic involvement (Nair et al., 2018). In addition, miR-122-5p, miR-132-3p, mir-1323, mir-136-5p, mir-182-3p, mir-210-3p, mir-29a-3p, mir-29b-3p, mir-342-3p, and mir-520h have significantly higher expression in GDM cases than in controls and have been reported to be involved in trophoblast proliferation, differentiation and insulin regulation and glucose transport in pregnant women (Figure 3E) (Gillet et al., 2019). A urine exosomal study in GDM patients in the third trimester of pregnancy revealed that miR-516-5p, miR-517-3p, miR-518-5p, miR-222-3p, and miR-16-5p are present in lower levels compared to a healthy pregnancy (Herrera-Van Oostdam et al., 2020). Increased level of exosomal dipeptidyl peptidase IV (DDPIV) is associated with GDM pathogenesis and a mice study showed that inhibitors of DDPIV inhibit glucose homeostasis by cleaving glucagon-like peptide 1. This could be used to treat type 2 diabetes (Figure 3E) (Kandzija et al., 2019). Thus, not only exosomes can serve as predictors for pathological pregnancy like GDM but can also be used as target molecules for the assessment of given therapeutics. Hyperglycaemic condition induces exosomes release in GDM pregnancy and interestingly these exosomes promote the release of pro-inflammatory cytokines like TNF-α, IL-6, GM-CSF, and IFN-γ from endothelial cells, thus contributing to the pathological inflammation in GDM (Salomon et al., 2016).

#### Preterm Birth

Studies on placental-derived exosomes in PTB are less and limited. Exosomes have been reported to carry miRNAs involved in the regulation of trophoblast invasion, proliferation and angiogenesis as potential biomarkers for predicting PTB such as hsamiR-381, hsa-miR-154, hsa-miR-377, and hsa-miR-150-5p (Figure 3F) (Menon et al., 2019; Cook et al., 2019). A set of proteins (FBLN1, IC1, F13A etc.) from plasma exosomes collected at 10–12 weeks of gestation are reported to be associated with the diagnosis of moderate PTB with the area under the receiver operating characteristic curve of 0.74 (Figure 3F) (McElrath et al., 2019). A comprehensive analysis of miRNA profiles of maternal plasma-derived exosomes differs at term and preterm and the miRNA’s target genes are associated with TGF-β signaling, p53, and glucocorticoid receptor signalling (Menon et al., 2019). A comprehensive proteomic profiling of PTB plasma-derived placental exosomal cargo has further verified that the alterations in protein compositions are also associated with inflammatory and metabolic signals. Interestingly, the placental senescence that occurs due to the encounter of oxidative and mitochondrial stress is reported to be influenced by these inflammatory signals (Figure 3F) (Cook et al., 2019). Studies performed on amniotic fluid-derived exosomes from preterm patients have confirmed these results (Dixon et al., 2018). A study in mice and cows demonstrated that *in-vitro* btamiR-499 found in pla-xosomes isolated from early pregnancy collected plasma, inhibited the activation of NF-κB via Lin28B/let-7 axis (lin 28B is an RNA Binding Protein and let7 is its targeted a miRNA) in bovine endometrial epithelial cells, suggesting that placental exosomes have a vital role in regulating uterine inflammatory balance determining a threshold for the onset of labor (Zhao et al., 2018b). *In-vivo* studies on mice have revealed labor-triggering properties of exosomes isolated from plasma of CD-1 mice from late gestation (E18) (Sheller-Miller et al., 2019). It emphasizes the importance of exosomal signals in the early termination of pregnancy.

### Similarities in the Development of the Placenta and Cancer

As pregnancy disorders involve the failure of feto-maternal cells to function normally, cancer begins with the failure of cells to reproduce and differentiate in a regulated manner. The development of the placenta and fetal-placental communication during pregnancy mimics a regulated form of cancer. Cancer manipulates the immune system for its survival in a similar manner as the placenta does for fetal survival. The cross-talk between cancer cells and immune cells is mediated via tumor exosomes (TEVs) (He et al., 2021). Interestingly, the cargo of TEVs also resembles similar to pla-xosomes indicating initiation of some similar pathways e.g., angiogenesis, T cell suppression, and expansion of anti-inflammatory responses during the growth spurt, later we will be exploring these aspects in detail. Expression of factors such as angiopoietins and members of the VEGF family occurs in placental and cancer development to aid in angiogenesis (Shore et al., 1997; Charnock-Jones et al., 2004). Therefore, a similarity can be drawn between the cellular invasion of EVT and cancer cells as early events in both the cases. Both of these cell types use the epithelial-to-mesenchymal transition to promote movement across the endometrium (during placental development) or normal (cancerous growth) tissue (Yang and Weinberg, 2008).

**FIGURE 4:**
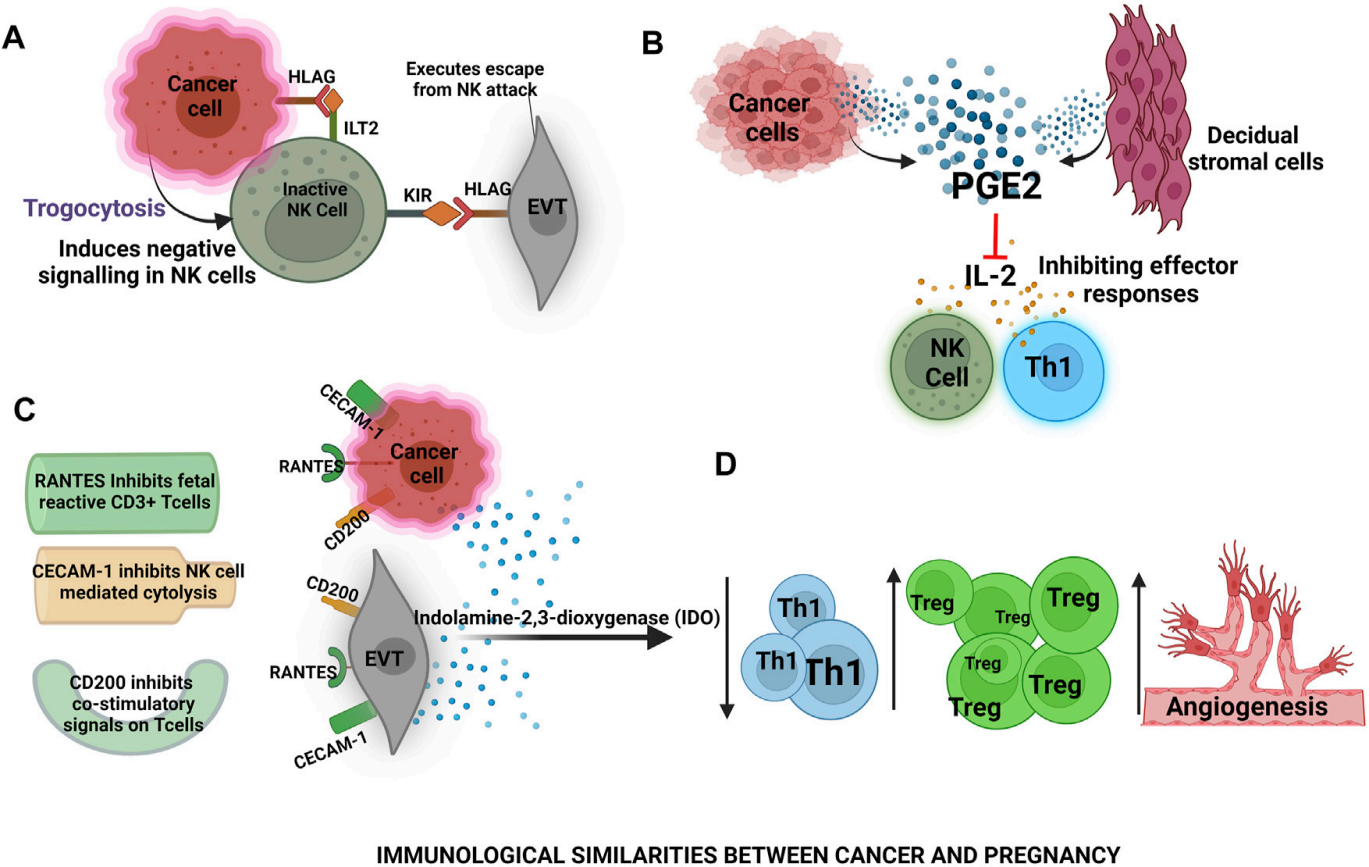
Immunological similarities between cancer and pregnancy- **(A)** In cancer, HLA-G causes suppression of NK cells activity and in placental development trophoblasts expressing the human leukocyte antigen G-5 (HLAG) induce killer inhibitory receptors on NK cells **(B)** Cancer cells and decidual stromal cells release prostaglandins that inhibit IL-2 production thus, masking pro-inflammatory responses by NK cells and T helper 1 cells **(C)** RANTES, CECAM-1, CD200 are the common surface molecules among cancer cells and extravillous trophoblasts (EVTs) that inhibit pro-inflammatory cellular responses **(D)** Cancer cells and EVTs secrete indolamine-2,3-dioxygenase (IDO) which is toxic to Th1 cells thereby ameliorating the Th1 expression and increasing regulatory T (Treg) cell responses to promote angiogenesis for the fetus and cancer survival.

Just like tumor cells are found in the systemic circulation, intact trophoblasts are also known to circulate in maternal peripheral blood during the early first trimester of pregnancy. Irrespective of HLA disparity these fetal-derived cells can embed in the maternal system establishing long-term microchimerism that persists for decades after parturition as a change accepted by the maternal immune system (Evans et al., 1999). Apart from the similar mechanism of development, the process for evading host immune response in cancer and trophoblast is also similar. Total or selective loss of HLA class I molecules is a frequently reported mechanism in various human tumors to escape recognition and destruction by cytotoxic T lymphocytes cells (Garcia-Lora et al., 2003). Trogocytosis (i.e., rapid cell-to-cell contacts that are dependent upon membrane transfer) is the primary mechanism by which HLAG + suppressive NK cells are generated within a tumor microenvironment (Caumartin et al., 2007). This mechanism is similar to HLA variants protection of trophoblasts in pregnancy where the trophoblast escape NK cell attack by inducing killer inhibitory receptors on NK cells reference from above (Figure 4A). Cancer cells also present the HLA class II antigen in the absence of the CD80/CD86 universe-stimulating molecules, this frequent representation of cancer cell antigens drives T-cell anergy thus, imparting cancer tolerance (Byrne and Halliday, 2003). Immune tolerance against cancer cells may also be the result of the knockout of lymphocyte lines that respond against autoantigens called tumour-associated antigens (TAA). These TAAs are abnormally expressed or overexpressed on malignant cells and is present in dissolved form in the circulation (Ko et al., 2003). Whereas, in the fetus, a combination of maternal and paternal antigens could contribute in chronic stimulation of T-cells thereby disrupting their effector functions. To ensure clearance from the immune system tumours are able to destroy immunocompetent T cells through a FasR/FasL-dependent mechanism causing T-cell apoptosis (Byrne and Halliday, 2003). A similar mechanism is executed by trophoblast cells for inducing T cell apoptosis. The tumor itself is resistant to Fas-mediated lysis by activated lymphocytes presumably because tumor cells overexpress BCL2 in the cytoplasm (Mese et al., 2000). Expressions of BCL2 have also been shown along the gestations in trophoblast cells however, contradicting studies revealed that expression of BCL2 is higher in the first and second trimester whereas, it has lower expressions in the third trimester of pregnancy emphasizing on the notion of pregnancy mirroring a regulated form of cancer which is a spatio-temporal need of the mother and the developing fetus (Soni et al., 2010). Just like fetal signals drive naïve T-cell differentiation into T regs, the tumor-specific antigens cause expansion of Treg cells in cancer implicating an impaired antitumor immunity, suppressed T cell proliferation, and increased tumor blood vessel density. This dampens the antitumor immune responses to promote angiogenesis (Beyer and Schultze, 2006). Immuno-regulatory mechanisms protect the fetus from the NK cell attack in the decidua. It was shown, Prostaglandin E2 (PGE2) (Figure 4B) which is derived from and localized in decidua aids in protecting the fetus by hindering the production of IL2 and the IL2 receptors on NK and T cells (Munn et al., 1998). This mechanism of host immune protection is hijacked by cancerous cells (Park et al., 2018). During pregnancy, membrane-bound and soluble molecules like LAG-3, Tim-3, PD-1, CTLA-4, and TIGIT are found which influence the Treg cell functions by decreasing the effectiveness of pro-inflammatory T cells (Zhang and Sun, 2020). Signals from cancer cells induce the expression of inhibitory receptor PD-1 on effector T cells setting them in a resting stage also known as T cell exhaustion. During the last decade PD-1, PD-L1 and CTLA-4 inhibitors have been used and were successful in aborting the solid tumours by setting the immune cells in their attacking state (Homet Moreno and Ribas, 2015; Robert, 2020). CD200 (OX-2) (Figure 4C) and carcinoembryonic antigen-related cell adhesion molecules (CEACAM-1), the cell surface tolerance signals exist commonly between trophoblasts and cancer cells (Clark et al., 2003; Gray-Owen and Blumberg, 2006). *In-vitro*, trophoblasts expressing CD200 can inhibit the generation of CD8^+^ T cells called cytotoxic lymphocytes (CTLs) and shift the balance of cytokines towards TH2 (Clark et al., 2003). CD200 in TME of melanomas, ovarian cancers, and renal cancers suppresses Th1 cytokines *in-vitro* (Moreaux et al., 2006). Inhibition of NK-mediated cytolysis also occurs by CEACAM-1 (CD66a), expressed on trophoblasts, whereas, CEACAM-1 in tumor cells diminishes expression of NKG2D receptors on NK cells, thus suppressing NK cytolysis implying another common link between cancer and pregnancy (Gray-Owen and Blumberg, 2006). A chemokine produced by trophoblasts known as RANTES is known to induce apoptosis of fetal-reactive CD3^+^ cells and the same chemokine is shown to be secreted by tumor-infiltrating lymphocytes following their apoptosis creating a mechanism for immune response evasion (Fraccaroli et al., 2009). Importantly, Indoleamine 2,3-dioxygenase (IDO) (Figure 4D) a tryptophan degrading enzyme is required for maintaining the tolerogenic state at the feto-maternal interface as well as in tumor microenvironment (TME) (Munn and Mellor, 2016). This enzyme converts tryptophan to kyneuirine, an effector T cell toxic compound inhibiting their proliferation and causing T cell apoptosis (Hwu et al., 2000). In a study performed on mouse models the action of enzyme IDO, when expressed at the interface of fetus and mother by MØ and trophoblast cells, was shown to be required for the protection of the semi-allogenic fetus. Moreover, the inhibition of IDO turned out cynical and lead to the death of the semi-allogeneic fetus (Munn et al., 1998). Whereas, IDO in TME, positively regulates the activity of Treg cells and this property has been used for the advantage of immunotherapy with IDO inhibitors (Yentz and Smith, 2018). In women with normal pregnancies, soluble CD30, a member of the tumor necrosis superfamily of receptors and a marker of TH2 polarization, is increased, while it is reduced in women with PE and intrauterine growth retardation (Figure 4D) (Kusanovic et al., 2007). Microarray analysis of placentae from pre-eclamptic pregnancies revealed changes in gene expression pathways including angiogenesis, immune defense responses as well as apoptosis, and cell survival which is also associated with cancer (Louwen et al., 2012).

### Cancer Escaping the Immune System: Unraveling the Trail of Cancer-Derived Exosomes (CEV_S_)

Pregnancy and cancer connect with each other at another aspect that is immunomodulation via exosomes. Studies have demonstrated the presence of similar signalling molecules (RNAs and/or proteins) encapsulated inside cancer-derived and placental-derived exosomes. Rigorous studies carried out in the field of cancer provide the initial understanding of the mechanistic pitfalls that may lead to pregnancy complications and adverse outcomes. The manipulation of host immune cells by cancer derived-exosomes to strengthen a tolerogenic milieu for the progression of cancer has been very well studied. This well-trodden path in the field of cancer biology can be tested using appropriate animal models and subsequent clinical trials to restore the lost tolerance and recreation of the anti-inflammatory milieu for the betterment of pregnancy complications. Therefore, it would be interesting to track the trail of cross-talk of cancer- and host immune cells via exosomes.

### CEVs Modulate Innate Immune Cells

**FIGURE 5:**
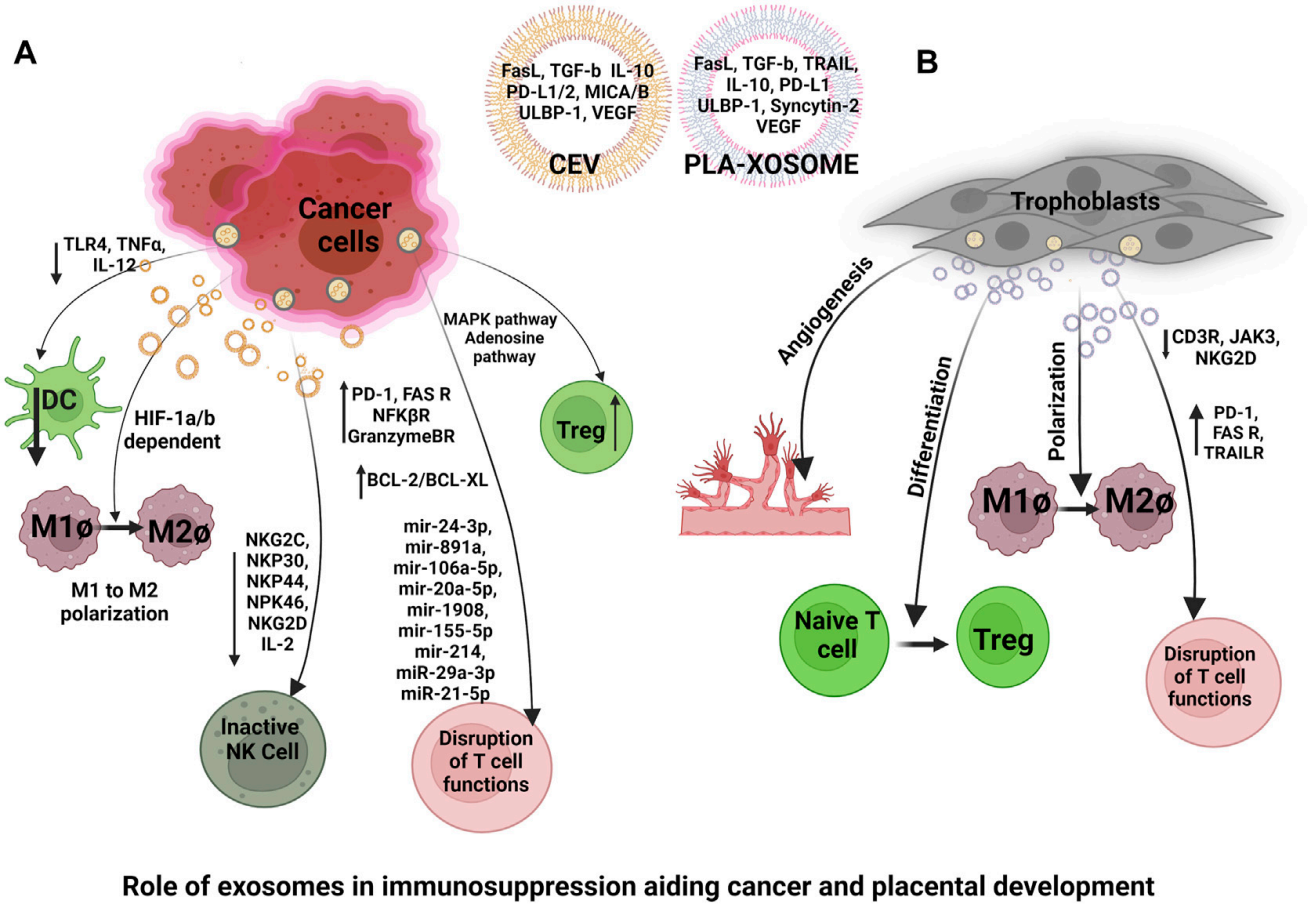
Targets of cancer-derived exosomes (CEVs) and placenta derived exosomes (Pla-xosomes)- **(A)** CEVs contains mir-203 which downregulates expression of toll-like receptor 4 (TLR4), downstream tumor necrosis factor-alpha (TNF-α) and interleukin-12 (IL-12) cytokines responsible for DCs proliferation and expansion, CEVs causes M1Ø to M2Ø polarization in a HIF-1a and HIF-1b dependent manner to promote immune suppression, CEVs internalization in NK cells, inhibits the expression of NK activating receptors like NKG2C, NKP30, NKP44, NPK46, and NKG2D to escape NK cell cytotoxicity, CEVs increases FasL/FasR signalling, PD-L1/PD-1 signalling and BCL2 (anti-apoptotic protein) expressions to evade apoptosis of cancer cells. CEVs carrying miR-29a-3p and miR-21-5p, miRNA 155-5p, miRNA-214, miR-24-3p, miR-891a, miR-106a-5p, miR-20a-5p, and miR-1908 inhibits T-cell activity, CEVs also cause T reg expansion thus aiding cancer development **(B)** Pla-xosomes promote angiogenesis via VEGF, help in Treg expansion, M2Ø polarization, causes upregulated expression of PD-L1, Fas, TRAIL and downregulates the expression of CD3 receptor, JAKR and NKG2D. All of these are essential to promote an effective immune microenvironment in the mother.

CEVs deviate the conventional pathway of the expansion of the myeloid and bone marrow precursor cells that are committed towards stimulatory DC into their suppressor phenotypes thus, altering the cancer antigen presentation via DCs and augmenting the tolerogenic niche (Ning et al., 2018; Tung et al., 2018). The miRNA-212 in pancreatic CEVs upon its internalization in DCs, downregulates the expression of transcription factor RFXAP (Regulatory factor X associated protein) which simultaneously demeanours the expression of MHC-II on DCs affecting the antigen presentation via these DCs (Ding et al., 2015). Moreover, CEVs interfere with the expression of co-stimulatory molecules like MHC‐II, CD80, CD86 on DCs and increase the expression of co-inhibitory receptors on DCs like PD-1. Thus, affecting the maturation and migration process of DCs and converting the existing DCs into suppressive phenotypes (Ludwig et al., 2018). Another *in vivo* study on pancreatic cancer reported that in DCs, CEVs affect their proliferation and expansion by down-regulating TLR4, downstream TNF-α, and IL-12 cytokines via miR-203 (Figure 5A) (Zhou et al., 2014). CEVs also modulate MØ, since mutation acquired abilities of cancer cells enable them to hijack M1 and re-engineer them into M2. The existence of M2 polarized state in malignant cancer forte, expressing functional Arg1, VEGF, and CD163, CD23, CD204, along with cytokines like IL-10, TGF-β, TGF- α, and chemokines including CCL16, CCL17, and CCL22, confirms a congenial M2 state (Cheng et al., 2019). Increased cancer growth creates a hypoxic environment, which results in the release of CEVs that polarizes M1 into M2 in a HIF-1α and HIF-1β dependent manner (Figure 5A) (Hood et al., 2011). Thus, CEVs manipulate M1 to exhibit M2 anti-inflammatory phenotype to help aid angiogenesis for fulfilling the oxygen demand of growing cancer. Interestingly, ovarian CEVs carrying miRNAs like mir-222-3p, have been shown to disrupt Treg/Th17 immune balance. They have been implicated in inducing M2 polarization via STAT-3 signal-dependent pathway thereby, increasing Treg and M2 expansion. Besides a decrease in the Th-17 cell population has been observed contributing to the anti-inflammatory cancer microenvironment (Ying et al., 2016). CEVs also have been reported to inhibit caspases involved in apoptosis and transfer a functional receptor tyrosine kinase initiating the monocyte MAPK pathway (Song et al., 2016). Thus, these altered MØ can then encourage angiogenesis and metastasis required for cancer progression. Another important innate immune subset, NK cells contain switches in the form of activating as well as inhibitory receptors. Apoptosis of cancer cells in prostate cancer and acute leukemia is prevented by CEVs internalization in NK cells, which inhibits the expression of NK activating receptors like NKG2C, NKP30, NKP44, NPK46, and NKG2D (Figure 5A) (Garcia-Iglesias et al., 2009). CEVs have also been shown to target the TGF-β pathway, TGF-β which exists as TGF-latency associated peptide (LAP) in CEVs when bound to integrin a6βV is activated and induces Smad phosphorylation subsequently reducing NKG2D expression thus preventing NK cell cytotoxicity (Szczepanski et al., 2011). In a mice model, CEVs treatment affected the generation of NK cells and also impaired their responses. CEVs encapsulate the stress-inducible NKG2D ligands, MHC-class I related protein chain A/B (MICA/B) and Ul-16 binding protein-1 (ULBP-1) and -2 that acts as a decoy, by down-regulating the NKG2D-mediated cytotoxicity of NK cells in T- and B-cell leukemia/lymphoma (Clayton and Tabi, 2005; Mincheva-Nilsson and Baranov, 2014b). In addition, CEVs suppressed the cyclinD3 expression and inactivate the JAK3 pathway by inhibiting IL-2 stimulation via NK cells thereby, breaking one connective link in innate and adaptive immunity by preventing T cell interaction with NK cells. Murine mammary carcinoma exosomes promote tumor growth by suppression of NK cell function (Liu et al., 2006). However, as disconnecting a single link cannot produce desirable results, thus CEVs interact with adaptive immune cells too.

### CEVs Modulate Adaptive Immune Cells

CEVs express CD39 (NTP-Dase) and CD73, which work together to convert extracellular ATP to immunosuppressive adenosine and 5 AMP phosphate (Clayton et al., 2011; Muller et al., 2016). Extracellular adenosine production is high, which adversely affects T cells around cancerous tissues, allowing it to evade immune responses. In addition, the presence of CEVs carrying miR-24-3p, miR-891a, miR-106a-5p, miR-20a-5p, and miR-1908 inhibits T-cell activity in nasopharyngeal cancer (Figure 5A) (Bell and Taylor, 2017; Ye et al., 2014). Interestingly, co-culturing CEVs with T cells resulted in elevated expression of BAX (proapoptotic marker) and decline in expression of BCL-2/BCL-XL (anti-apoptotic markers) indicating cancer mediated T cell suppression (Figure 5A). FasL in CEVs causes the apoptosis of FasR + T cells by initiating FasL/FasR signalling (Alzahrani et al., 2018). According to an analysis of EVs recovered from the serum of patients with head and neck cancer and melanoma, cell death ligands such as Fas on CD8^+^ cytotoxic T lymphocytes (CTLs), were particularly sensitive to CEVs. They affected signal transduction and proliferation of CD8^+^ CTLs thus, affecting cytotoxic responses on cancer cells (Maybruck et al., 2017). Peritoneal tissue from patients with metastatic ovarian cancer had higher Treg levels than Th17 cells suggesting a requirement of more suppressed TME for metastasis (Zhou et al., 2018). It was found that exosomes play a unique role in this imbalance. Favoring T reg functions, exosomes originating from TAMs transfer miR-29a-3p and miR-21-5p to helper T cells and inhibit intracellular STAT3 signalling which decreases pro-inflammatory cytokine secretion from CD4^+^ T cells (Figure 5A). This disturbs the Tregs/Th17 balance creating an immunosuppressive environment for ovarian cancer progression (Zhou et al., 2018). In addition, there have been recent reports of CEVs containing PD-L1, which inhibit the immune system by targeting multiple pathways, thus aiding cancer growth (Figure 5A) (Mrizak et al., 2015). The transfer of PD-L1 via CEV from PD-L1_high_ cancer cells to PD-L1_low_ cancer cells elevated the PD-L1 release which further inhibited the T cell response by initiating PD-L1/PD-1 signalling. The membrane-bound PD-L1 carried by exosomes suppresses anti-cancer immune responses both locally in the TME and systemically. PD-L1+ exosomes produced by a breast cancer cell line inhibited co-stimulatory molecule (CD3/CD28) -induced ERK phosphorylation and NFκB activation of T-cells *in vitro*. Exosomal PD-L1 harbors active defense function to suppress T cell killing of breast cancer cells and promote tumor growth (Yang et al., 2018). The experiment carried out *in-vivo* revealed suppression of granzyme B activity of T cells found in the TME, thus reducing cytotoxic T-cell activity (Vignard et al., 2020). In another study, exosomes isolated from head and HNSCC patients’ plasma inhibited the activatory receptor CD69 expression on human activated CD8^+^ T cells, and the PD-L1 levels on exosomes correlated with their T-cell inhibitory activity. Murine CEVs carrying PD-L1 were immunosuppressive, and blocking of PD-L1 activity with neutralizing mAbs restored the immune competence of T cells and inhibited tumor growth (Theodoraki et al., 2018). CEVs caused the expansion of Tregs. Tregs are one of the most important subsets of T-cells required for sustaining the development and growth of biological entities. Secretion of anti-inflammatory cytokines like IL‐10, TGFβ-1, and CTLA4 promotes the suppressive phenotype of Treg which is immensely exploited by cancer cells. Researchers have confirmed the transformation and proliferation of CD4^+^CD25^+^ T-cells into CD4^+^CD25 + Foxp3+ Tregs *in-vivo* upon administration of CEVs via MAPK pathway and adenosine pathway (Figure 5A) (Mrizak et al., 2015) miRNA-155-5p and miRNA-214 in CEV inhibited the precursor T-cell differentiation into Th1/Th17 phenotypes and reduces the PTEN-tumor suppressor homolog in T cells respectively, therefore increasing anti-inflammation and decreasing pro-inflammation parallelly (Figure 5A) (Yao et al., 2012; Sharma et al., 2015; Sun et al., 2019). *In-vitro*, CEV’s surface markers CD39 + CD73^+^ (NTPDases) bind to the T cell surface adenosine receptor 2 (A2AR) and send out a signal via cAMP. This upregulates the T cells to generate adenosine and prime Tregs thereby inducing their effector responses (Clayton et al., 2011). The elevated content of CD39/CD73 in CEVs reflected the presence of advanced-stage disease in HNSCC patients. These studies give strong evidence of impaired host immune response directed via CEVs (Allard et al., 2017). Interestingly, analyzing T cell-derived exosomes from cancer patient’s plasma for clues of the immune status in CEVs-reprogrammed T cells has recently become possible. Chimeric antigen receptor (CAR+) exosomes derived from CAR-T cells administered in cancer patients are enriched in immunosuppressive proteins and consistently inhibit functions of other T cells, thus their internalization causes intracellular changes in T cells (Fu et al., 2019).

### Therapeutic Potential of Exosomes in Pregnancy Complications

**FIGURE 6:**
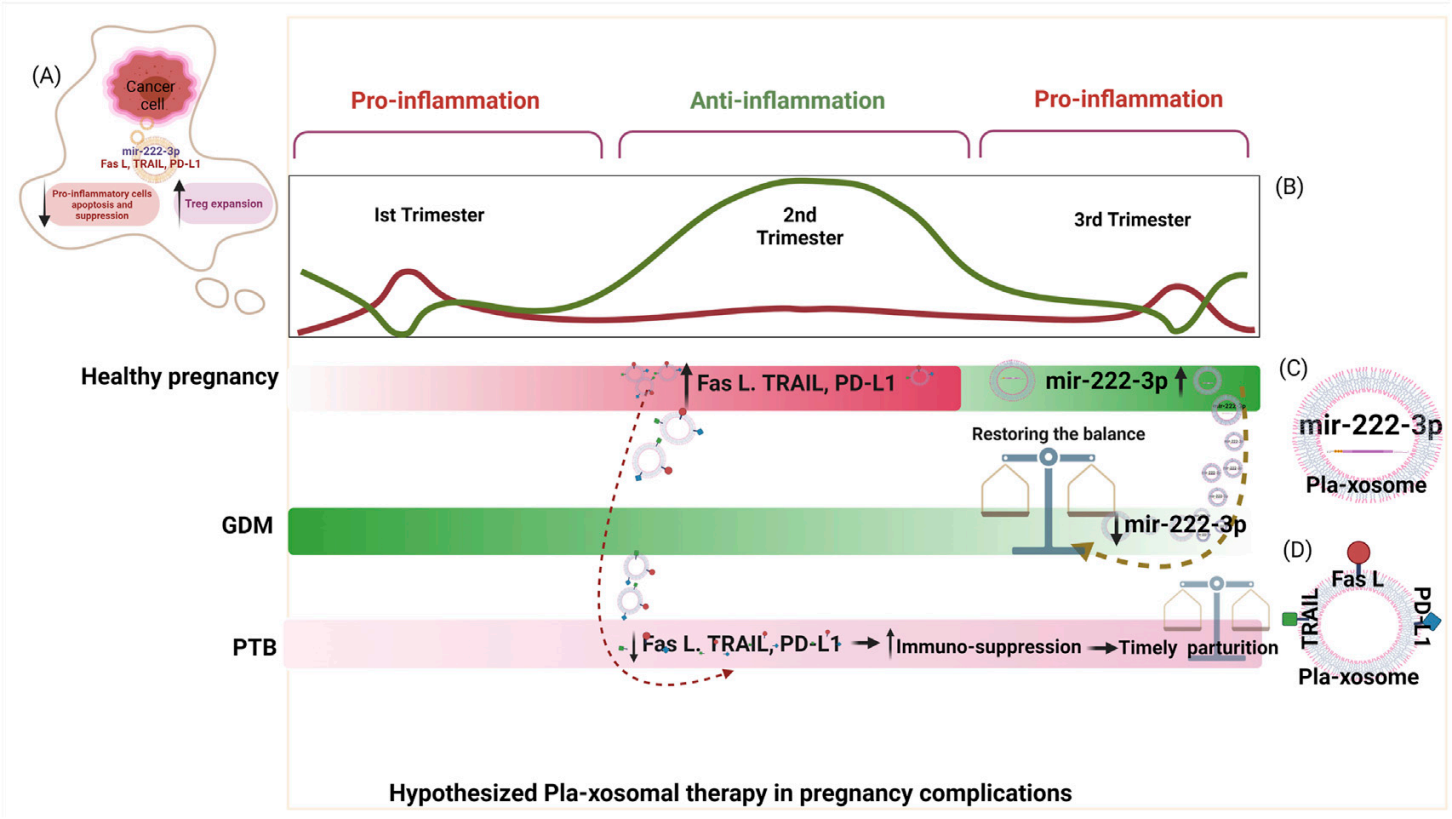
Hypothesized pla-xosomal therapy for pregnancy complications: Taking lessons from cancer **(A)** Cancer cells derived exosomes contain mir-222-3p, PD-L1, Fas L, and TRAIL that cause suppression of pro-inflammatory cells and expansion of anti-inflammatory cells (Tregs) **(B)** Balanced pro-and anti-inflammatory milieu in a healthy pregnancy with increased expression of PD-L1, Fas L, TRAIL in second Trimester and mir-222-3p in third Trimester **(C)** Pla-xosome containing mir-223: postulated therapy for GDM **(D)** Pla-xosome containing PD-L1, Fas, TRAIL: postulated therapy for PTB. Dotted lines represent the donor and recipient gestational windows of plax-osomes intervention.

The role of exosomes in cancer diagnosis and immune therapy has been extensively studied. As mentioned previously, cancer cells release PD-L1+ exosomes that interact with T cell’s surface PD-1 initiating intracellular suppressive signalling. In the advanced stages of cancer expression levels of soluble PD-L1 are increased that can be detected in circulation thus, cancer-derived exosomal PD-L1 can serve as cancer predicting biomarker (Figure 6A) (Shimada et al., 2021). Even for cancer therapy, the immune checkpoints are known targets for inhibitory antibodies. Moreover, the use of human umbilical cord blood mesenchymal stem cells-derived exosomal mir-503-3p has been reported to abort endometrial cancer and target biological functions of endometrial cancer cells by downregulating mesoderm-specific transcript (Pan et al., 2022). However, the use of exosomes for providing therapies in pregnancy complications is a big challenge because of the need for a balanced treatment at a particular time, simultaneously protecting the fetus from any harm. Irrespective of the challenges, multiple trials for creating therapeutics in restoring the balance of healthy pregnancy processes in pregnancy complications have been attempted. For e.g., in a mouse model study, exosomes from human umbilical cord mesenchymal stem cell-derived (HUMSC) exosomes have been reported to improve endometrial injury by stimulating endometrial regeneration via PTEN/AKT signalling pathway. This further increases the expression of BCL-2 (anti-apoptotic protein) via AKT activation and decreases the expression of activated caspase-3 facilitating cell proliferation thus promoting endometrial regeneration (Wang et al., 2020b). Another study demonstrated that the administration of HUCMSC exosomes results in upregulation of mir-18b-3p, which targets leptin to reduce pro-inflammatory factors and prevent cellular apoptosis in the PE rat placenta (Huang et al., 2021). Interestingly in the mouse model of PE, the therapeutic effects of HUCMSCs derived EVs have been reported where administration of HUCMSC-exos during pregnancy prevented soluble Fms-like Tyrosine kinase (sFLT-1) induce preeclamptic complications. sFLT is a negative regulator of VEGF thus aiding angiogenesis, HUMSCs-exos input resulted in decreased sFLT levels thereby, ultimately improving the fetal and placental weight. The exosomes have engineered to encapsulate IκBa that inhibit pro-inflammatory cytokine transcription factor NFkB in feto-maternal uterine tissues thus, delaying LPS-induced PTB (Sheller-Miller et al., 2021). Administration of mesenchymal stromal cell-derived extracellular vesicles alters inflammatory mediators’ expression in the preeclamptic intrauterine compartment, thus normalizing the formation of fetal lung branches and their morphogenetic gene expressions (Taglauer et al., 2021).

### Taking Lessons From CEVs

Due to the uncanny resemblance of the underlying biological processes of pregnancy with cancer, the signal carrying exosomal cargo in both are also close to similar. The immunosuppressive entities harbored in the exosomes e.g., PD-L1, VEGF, MICA, ULBP-1, HLA variants, Fas L, TRAIL, IL-10 etc. target similar immune cell subsets like Th1, Tregs, DCs and NK cells thus, promoting the anti-inflammatory niche required for the fetus and cancer development post its implantation and establishment respectively (Figure 6B). Interestingly, ovarian-cancer-derived exosomes contain mir-222-3p that is shown to increase Tregs thus, promoting anti-inflammation required for cancer survival (Stenqvist et al., 2013). Whereas, in GDM patients the expression of placental derived exosomal mir-222-3p significantly decreases by the third trimester and affects the metabolic processes like steroid hormone biosynthesis and tryptophan metabolism triggering insulin resistance and inflammation in GDM (Herrera-Van Oostdam et al., 2020). However, as a healthy pregnancy progresses, elevated levels of mir-222-3p have been observed, implying that the increased expression of this miRNA is a requirement for an uncomplicated pregnancy (Herrera-Van Oostdam et al., 2020). Since the mir-222-3p is enriched within placental exosomes, these exosomes could be used in a spatio-temporal manner to ameliorate pregnancy complications like GDM as a therapeutic agent (Figure 6C). Similarly, the apoptosis-inducing ligands like FasL, TRAIL, and immune exhaustion markers like PD-L1 are enriched on pla-xosomes and their isolation would be more appropriate from the second trimester of a pregnancy where an anti-inflammatory milieu is a necessity for fetal development (Figure 6D) (Stenqvist et al., 2013). These pla-xosomes may be used as therapeutics for treatment in pregnancy complications like PTB where inflammatory responses are high causing early parturition. However, isolation and delivery of these power shots should be carried out in a timely manner i.e., pla-xosmes isolated from the second trimester of a healthy pregnancy need to be administered to a high-risk mother diagnosed for preterm delivery so as to decrease the inflammation and lengthen the gestational age *in utero*. However, for such a successful execution of this hypothesized therapy, the identification of early predictive markers for adverse pregnancies is an obligation and clinical trials are vital.
